# Deciphering the Molecular Mechanism of Substrate-Induced Assembly of Gold Nanocube Arrays toward an Accelerated Electrocatalytic Effect Employing Heterogeneous Diffusion Field Confinement

**DOI:** 10.1021/acs.langmuir.2c01001

**Published:** 2022-07-27

**Authors:** Pawel Niedzialkowski, Adrian Koterwa, Adrian Olejnik, Artur Zielinski, Karolina Gornicka, Mateusz Brodowski, Robert Bogdanowicz, Jacek Ryl

**Affiliations:** † Department of Analytic Chemistry, University of Gdańsk, Wita Stwosza 63, 80-952 Gdańsk, Poland; ‡ Department of Metrology and Optoelectronics, Faculty of Electronics, Telecommunications and Informatics, Gdańsk University of Technology, Narutowicza 11/12, 80-233 Gdańsk, Poland; § Centre for Plasma and Laser Engineering, The Szewalski Institute of Fluid-Flow Machinery, Polish Academy of Sciences, Fiszera 14, 80-231 Gdańsk, Poland; ∥ Department of Electrochemistry, Corrosion and Materials Engineering, Gdańsk University of Technology, Narutowicza 11/12, 80-233 Gdańsk, Poland; ⊥ Institute of Nanotechnology and Materials Engineering and Advanced Materials Center, Gdańsk University of Technology, Narutowicza 11/12, 80-233 Gdańsk, Poland

## Abstract

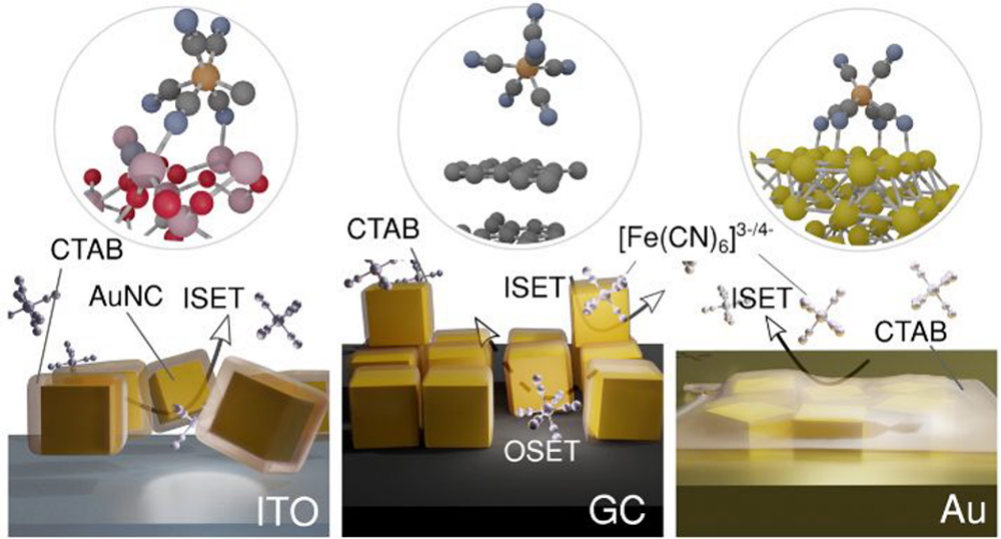

The complex electrocatalytic performance of gold nanocubes (AuNCs) is the focus of this work. The faceted shapes of AuNCs and the individual assembly processes at the electrode surfaces define the heterogeneous conditions for the purpose of electrocatalytic processes. Topographic and electron imaging demonstrated slightly rounded AuNC (average of 38 nm) assemblies with sizes of ≤1 μm, where the dominating patterns are (111) and (200) crystallographic planes. The AuNCs significantly impact the electrochemical performance of the investigated electrode [indium–tin oxide (ITO), glassy carbon (GC), and bulk gold] systems driven by surface electrons promoting the catalytic effect. Cyclic voltammetry in combination with scanning electrochemical microscopy allowed us to decipher the molecular mechanism of substrate-induced electrostatic assembly of gold nanocube arrays, revealing that the accelerated electrocatalytic effect should be attributed to the confinement of the heterogeneous diffusion fields with tremendous electrochemically active surface area variations. AuNC drop-casting at ITO, GC, and Au led to various mechanisms of heterogeneous charge transfer; only in the case of GC did the decoration significantly increase the electrochemically active surface area (EASA) and ferrocyanide redox kinetics. For ITO and Au substrates, AuNC drop-casting decreases system dimensionality rather than increasing the EASA, where Au–Au self-diffusion was also observed. Interactions of the gold, ITO, and GC surfaces with themselves and with surfactant CTAB and ferrocyanide molecules were investigated using density functional theory.

## 1 Introduction

Gold nanoparticles (AuNPs) possess many excellent properties as a consequence of their shapes, sizes, and molecular structures. These parameters mainly affect their biocompatibility,^1^ chemical stability, and optical and electrical properties,^2^ which mainly determine their practical application as catalysts,^3,4^ as sensors, and for nanotechnology and biomedical purposes.^5^ Additionally, the properties of AuNPs are determined by further modifications with either organic species or hybrid or inorganic nanomaterials. The possibility of their modification with amines,^6^ phosphine,^7^ and thiol derivatives^8,9^ has a remarkable influence on the practical application of AuNPs for biosensing^10^ and many biomedical applications such as cancer treatment,^11,12^ drug delivery,^13^ optoelectronic devices and bioimaging,^14^ and therapeutics.^15^ There is broad interest in the recognition of electrochemical and physicochemical surface properties after deposition and functionalization with various gold nanostructures. The application of AuNPs is mainly focused on biosensors and electrocatalysis. Among many notable examples, functionalized AuNP/Au systems were used for sensitive microRNA detection and enzymatic amplification^16^ and bacterial lipopolysaccharide recognition.^17^ The assessment of tau proteins in Alzheimer’s patients was also possible with AuNP structured at screen-printed carbon electrodes.^18^ Molecular tethering of AuNPs at boron-doped diamond^19^ or GC^20^ offers versatility through its electrocatalytic behavior and reproducible responses. Oxide electrodes are commonly modified by AuNPs with the aim of providing an easy alternative for their surface modification. Such an approach was reported for the detection of oral cancer salivary biomarker interleukin-8 (IL8) using AuNPs-rGO/ITO^21^ and non-enzymatic sensors for methyl parathion.^22^ An antigalvanic replacement by surface-confined AuNPs revealed the size-dependent catalytic properties.^23^

The electrochemical activity of nanoparticles depends on the abilities of the surface electrons to promote a catalytic process. Therefore, the electrical and electrocatalytic properties of AuNPs are directly connected with their structure, size, or crystallographic orientation.^24^ Many different synthetic approaches have been invented to obtain nanoparticles consisting of regular or irregular shapes, influencing the surface atom densities, physical properties, electronic structure, and chemical activity. Currently, there are several methods known for the synthesis of nanoparticles resulting in regular shapes of various morphologies such as cubes, rods, spikes, hexagonal or triangular plates, cages, and pyramids.^11,25,26^ On the basis of their electrocatalytic activities toward glucose oxidation, it has been shown that gold nanocrystals possess different activities. For gold nanobelts and nanoplates, it has been found that the (110) surface of gold is more active than the (111) surface for glucose oxidation, while for methanol oxidation in an alkaline solution, the activity of the (110) surface is lower than that of the (111) surface.^24,27^ Additionally, it has been proved that Au nanocrystals bound with (100) surfaces are significantly more active than (110)-bound rhombic dodecahedral and (111)-bound octahedral Au nanocrystals toward glucose oxidation.^28^ In the case of gold nanorods, glucose oxidation is enhanced in the presence of (100) facets.^29^ The presence of ions or surfactants during the synthesis influences the structure of the obtained nanoparticles, which directly influences the electrochemical properties. For instance, the presence of iodine during the synthesis results in a higher ratio of (100) and (111) facets in the presence of sulfides.^30^ Hexadecyltrimethylammonium bromide (CTAB) is commonly used in the synthesis of nanoparticles in the seed-mediated methods. The choice of CTAB for synthesis makes it possible to obtain large single-crystal nanoparticles.^25^ Furthermore, a specific shape and a specific crystallographic orientation of AuNPs are possible upon adsorption of CTAB, because it interacts differently with gold facets in the (100) ≈ (110) > (111) order.^24^ The presence of a surfactant will further influence the electrocatalytic activity of the AuNPs.^31^

In recent years, gold nanocubes (AuNCs) have been of great interest due to their three-dimensional structure. In addition to the fact that all AuNCs possess the same dimensions, each can form more regular or random forms, self-organizing more predictably, which strengthens the attraction to this material. The major advantage of AuNCs in comparison to AuNPs is their chemical stability, the possibility of further functionalization, and their unique tunable plasmonic properties. In addition, single AuNCs or their agglomerates can cause amplification of electromagnetic fields interacting with neighboring particles, enhancing their activity, which allows these particles to be used in the creation of optical sensors.^32^ Gold cubic nanoparticles were also investigated with respect to the oxygen reduction reaction deposited on a disc electrode.^33^ The unusual properties resulting from the regular shape of the AuNCs allow the application of localized surface plasmon resonance (LSPR) techniques for detecting biomolecules.^34,35^ Today, AuNCs may be used for the preparation of label-free electrochemical aptasensors and immunosensors. AuNCs were deposited on a gold substrate in a multistage modification with cysteamine and, after aptamer immobilization, used for sensitive and selective chloramphenicol detection.^36^ Lv et al. used AuNCs deposited on graphene oxide to create a sandwich immunoassay for the detection of a cardiac biomarker (troponin). The aim of using AuNCs was to immobilize the antibody and accelerate the electron transfer through the interface.^37^

There is no description of the mechanism of interaction of AuNPs with various common electrode substrates in the literature. AuNPs affect the electrochemical activity of the studied systems to different degrees due to the ability of surface electrons to promote the catalytic processes.^12,38^ The regular shapes with defined facets of AuNCs and the ease of their specific self-organization at the electrode surface offer desirable conditions for the determination of electrocatalytic processes. To the best of our knowledge, no research has focused on evaluation of the self-organization of AuNCs at common electrode surfaces. Therefore, it is very important to improve our understanding of those interactions, which often remains unclear. Herein, we present fresh insight into the interaction of AuNCs with the three most commonly used transmitters for electrochemical sensors, namely, glassy carbon (GC), indium–tin oxide (ITO), and gold. We discuss the enhancement of the electrochemical activity and the complexity of the electrode/electrolyte interface, which are affected by AuNCs’ three-dimensional self-assembly at the electrode substrates through different molecular interactions, the appearance of the coffee-ring effect during the drop-casting procedure,^39,40^ and the non-negligible impact of the surfactant (CTAB) and different electron transfer pathways (tunneling and bridging) on the electroactive compounds.

## 2 Experimental Section

### 2.1 AuNC Synthesis and Deposition

All chemicals were of analytical grade and used as received without further purification. Cetyltrimethylammonium bromide (CTAB), sodium borohydride (NaBH_4_), potassium hexacyanoferrate(III) [K_3_Fe(CN)_6_], and potassium hexacyanoferrate(II) [K_4_Fe(CN)_6_] were purchased from Sigma-Aldrich. Phosphate-buffered saline (PBS) buffer from ThermoFisher Scientific was obtained by dissolving tablets in deionized water. Ascorbic acid (AA) and hydrogen tetrachloroaurate(III) (HAuCl_4_·4H_2_O) were obtained from POCH.

A two-step seeding growth procedure was used to synthesize Au nanocubes according to the modified procedure described previously.^36,41^ The first step involved the synthesis of Au seeds. First, 0.3 mL of 0.01 M NaBH_4_ was added in dropwise fashion over 5 min to a flask containing a mixture of 3.75 mL of 0.1 M CTAB and 0.125 mL of 0.01 M HAuCl_4_·4H_2_O, under stirring. Then, the obtained solution was stirred at room temperature for 12 h.

**Figure 1:**
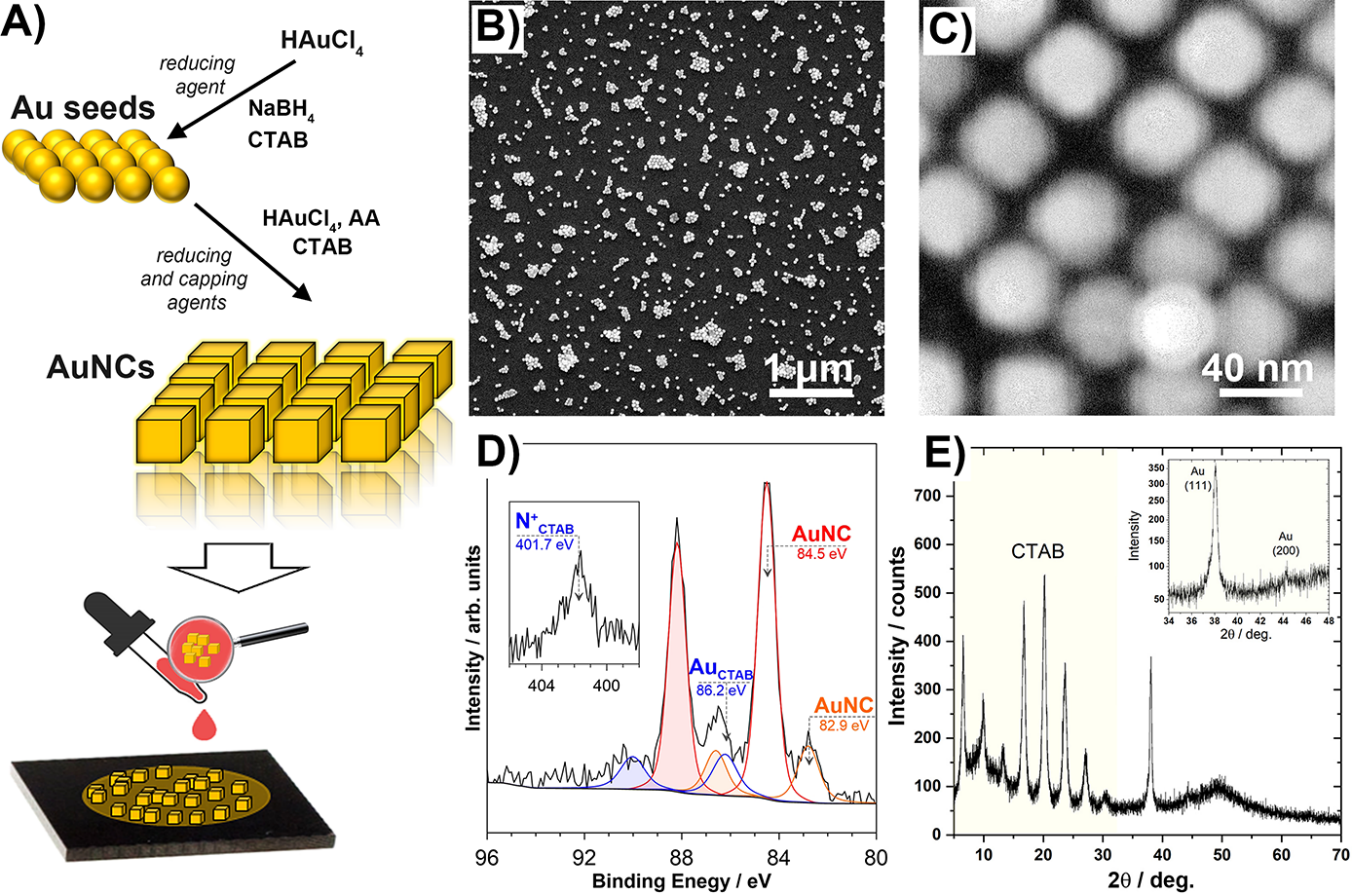
(A) Schematic presentation of AuNC synthesis and application. (B and C) SEM images of the AuNCs decorated at the GC surface. (D) AuNC/GC system XPS analysis in the Au 4f core-level energy range, with the N 1s spectrum shown in the inset. (E) X-ray diffraction pattern for AuNC. The inset shows the (111) and (200) reflections of a gold phase.

In the next step, the AuNCs’ growth solution was obtained by mixing 6.4 mL of 0.1 M CTAB, 0.8 mL of 0.01 M HAuCl_4_·4H_2_O, and 3.8 mL of 0.1 M AA. Then 30 μL of Au seeds was added, diluted 10-fold, to the growth solution. After the mixture had been vortex mixed for 30 s, the obtained solution was left overnight. After 24 h, the AuNCs were centrifuged (5000 rpm, 20 min) and washed twice with a 0.001 M CTAB solution. The scheme of this procedure is simplified in Figure 1A. The AuNCs were stored in a 0.001 M CTAB solution and used for further investigations.

Electrode surface modification was performed by dropping different amounts of AuNCs of suspended 0.001 M CTAB from 10 to 50 μL on the surface of GC, ITO, and Au electrodes. The GC and Au electrodes were first polished with 50 nm aluminum oxide on a polishing cloth, cleaned with water, and dried under a stream of nitrogen. Then, 10 μL of the AuNC solution was drop-cast on the electrode surface and left to dry in the air for 30 min at room temperature. The electrodes with deposited AuNCs were washed twice with water before use. The same modification procedure was applied for the ITO electrode previously cleaned by ultrasonication in methanol for 5 min and dried in air.

### 2.2 Electrochemical Studies

All electrochemical measurements were performed on an Autolab M204 potentiostat (Metrohm) using a three-electrode system. The working electrode was GC or a thin ITO film on glass, while a thin Au film on glass or Ag/AgCl (3 M KCl) was used as the reference electrode; a platinum wire was used as the counter electrode. All potentials are presented versus the reference electrode. The electrochemical cell volume was 5 mL, and the electrolyte-exposed electrode surface area was 0.20 cm^2^.

Cyclic voltammetry (CV) measurements were conducted in a solution consisting of equimolar amounts of 1 mM K_3_[Fe(CN)_6_] and K_4_[Fe(CN)_6_] dissolved in 0.01 M PBS (pH 7.4). All cyclic voltammograms were recorded in the potential range of −0.35 to 0.60 V with a scan rate from 0.5 to 500 mV s^–1^. The electrochemically active surface area (EASA) for the investigated electrodes was calculated by the Randles–Sevcik equation (R–S). The CV measurements were also performed in 0.1 M H_2_SO_4_ as the electrolyte solution in the potential range of −0.10 to 1.50 V with a scan rate of 20 mV s^–1^. The electrochemical impedance spectroscopy (EIS) experiment was carried out under open circuit potential (OCP) conditions with a voltage perturbation amplitude of 10 mV and a frequency range from 100 kHz to 0.1 Hz, with 40 points per frequency decade.

Scanning electrochemical microscopy (SECM) measurements were performed using a commercially available positioning setup (Sensolytics GmbH). It consists of three stepper motor-controlled precision linear stages that allow a maximum travel range of 25 mm × 25 mm × 25 mm with a step width of 20 nm. Additionally, the positioning system contains a piezoelectric system (P-611.3 NanoCube XYZ Piezo System, Physik Instrumente GmbH & Co. KG) that provides a travel range of 100 μm × 100 μm × 100 μm with a resolution of 1 nm. The positioning system was combined with an Autolab PGSTAT302N potentiostat (Metrohm) with an additional ECD module for low-current measurements. SECM measurements were performed in the three-electrode configuration consisting of a gold microelectrode (active electrode area with a 10 μm diameter, RG ratio of 20) as the working electrode, a platinum wire as the counter electrode, and a Ag/AgCl gel electrode as the reference electrode. All of the electrodes were purchased from Sensolytics GmbH. Before the measurements, the microelectrode was polished using alumina oxide and characterized via CV. The electrolyte utilized during the measurements was 5 mM K_3_[Fe(CN)_6_] dissolved in PBS (pH 7.4) and deoxygenated prior to experiments. The reduction of [Fe(CN)_6_]^3–^ was carried out at −0.2 V versus Ag/AgCl (gel). The working distance was established by touching the sample’s surface. All experiments were performed in feedback mode (unbiased investigated sample). The approach curves were registered from ∼10 radii of a utilized microelectrode using a piezo positioning system with a step of 100 nm and a speed of 1 μm s^–1^ until the touching point was observed. The illustrative areas of SECM and AFM analyses are presented in section S1 of the Supporting Information.

### 2.3 Physicochemical Studies

Scanning electron microscopy (SEM) studies were used to analyze the AuNC distribution. The measurements were carried out using an FEI Quanta 250 FEG instrument (ThermoFisher Scientific) equipped with a Schottky field emission gun, operating at an accelerating voltage of 30 kV.

The X-ray diffraction (XRD) measurement was carried out at room temperature on a Bruker D2 Phaser diffractometer with Cu Kα radiation (λ = 1.54056 Å) and an XE-T detector. The data were collected for the 2Θ range of 5–70°. For the AuNCs, the measurement was performed on the silicon plate.

AFM topographic measurements were taken using a NTegra II device produced by NT-MDT Corp. Imaging was performed under atmospheric conditions but with the use of mechanical and electrical insulation with a protective cover. Images were taken sequentially for areas ranging from 10 to 2 μm to identify and magnify the surface structures observed. Imaging was performed in semicontact mode, with a set point equal to half the amplitude of free oscillation. NSG30 probes by NT-MDT were used for the measurements dedicated to semicontact measurements. Cantilever geometrical parameters were 125 μm (*L*) × 40 μm (*W*) × 4 μm (*T*). The measurement frequency was 296 kHz.

X-ray photoelectron spectroscopy (XPS) studies were carried out using an Escalab 250Xi instrument (ThermoFisher Scientific), operating with an AlKα source. The X-ray spot diameter was 650 μm, and the pass energy was 20 eV. The low-energy electron and Ar^+^ ion flow served charge compensation purposes, with final calibration of the spectra on adventitious carbon C 1s (284.8 eV).

### 2.4 Density Functional Theory (DFT) Calculations

The surface and molecular structures were designed using a builder tool provided by Atomistic ToolKit Quantumwise (ATK, Synopsys) as reported in ref (42). Interactions of the gold, ITO, and GC surfaces with themselves and with the CTAB and ferrocyanide molecules were investigated by performing geometry optimizations and calculating electron density maps on the optimized structures. Surface calculations were performed using slabs (flat surfaces) of cubic Au (100), hexagonal graphitic carbon (001), and cubic ITO model cleaved across the (100) plane. DFT at the generalized gradient approximation (GGA) level of the theory with the Perdew–Burke–Ernzerhof (PBE) functional was applied as implemented in the package for most calculations. Application of DFT-D3 correction for van der Waals interactions is indicated in the text.^43^ The linear combination of atomic orbitals (LCAO) method^44^ with PseudoDojo norm-conserving pseudopotentials and the medium ATK basis set were applied.^45^

The ferrocyanide and CTAB adsorption phenomena were investigated only on flat surfaces with slab models with the size appropriate for the DFT level of theory. Gibbs free energies of CTAB adsorption were calculated according to eq 1:
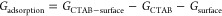
1An analogous equation was used for adsorption of ferrocyanide ions on the unoccupied surface. The energies of adsorption on the CTAB-occupied surfaces, however, were calculated according to eq 2:
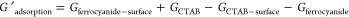
2The disturbance energy is defined as the difference between *G*_adsorption_ and *G*′_adsorption_.

## 3 Results and Discussion

### 3.1 AuNC Characterization

The topography of the obtained AuNCs is presented in panels B and C of Figure 1. Moreover, Figure 1B demonstrates the homogeneous spatial distribution of AuNCs at the GC surface using the proposed drop-cast method. Some of the AuNCs form larger clusters reaching up to ∼500 nm in size. On the basis of the micrographs taken in the center of the drop-cast area, the thickness of these clusters typically does not exceed two AuNC layers. A great majority of the AuNCs are slightly rounded cube-like polyhedra with a uniform size of 38 ± 2 nm.

High-resolution XPS analysis of the AuNCs drop-cast at the GC substrate was performed in the Au 4f core-level binding energy (BE) range. The studies revealed a complex spectral structure that was deconvoluted using three components (Figure 1D). The major Au 4f_7/2_ component (70% [Au]) was found at 84.5 eV, the energy typically reported for gold nanoparticles^46,47^ and thus ascribed to AuNCs. With a [Au] share of 13%, the next peak is shifted by +1.7 eV and originates from oxidized Au(III) complexed by CTAB.^48,49^ The presence of a stabilizing agent was further confirmed by the N 1s signal of the pure ligand (Figure 1D, inset).^50^ Determination of the origin of the third Au 4f component is the most problematic. Its share within [Au] is roughly 17%. Passiu et al.^47^ recognized the appearance of a −0.4 eV-shifted surface component, yet in our case, the peak is shifted by −1.6 eV versus the main component. The chemistry of AuNCs decorated at Au and ITO substrates revealed similar chemistry, but altered shares of individual components, as deconvoluted in section S2 of the Supporting Information. These analyses suggest a significantly higher level of Au(III) species complexed by CTAB compared to the GC substrate and hint at different mechanisms of the AuNC–electrode interaction.

The XRD pattern of the AuNCs is presented in Figure 1E. The first and highest reflection at (111) for Au is observed near a 2Θ of 38°, in agreement with the ASTM powder diffraction data. The diffraction peaks observed for the 2Θ range of 5–35° are attributed to the CTAB.^51^ The ideal nanocubes should be predominately enclosed by (200) facets.^52^ On the contrary, different AuNC synthesis approaches, including ours, lead to the formation of cube-like polyhedra with (111) and (200) crystallographic planes dominant in the XRD patterns.^53,54^ For a randomly oriented polycrystalline gold powder, the theoretical value of the *I*_(200)_/*I*_(111)_ ratio is 0.5,^55^ while in our case, the *I*_(200)_/*I*_(111)_ ratio is 0.1. This suggests that crystallites in AuNCs are oriented along the (111) direction, in accordance with the CTAB interaction propensity with major Au crystallographic planes.^24^ The average crystallite size was estimated from the Scherrer formula on the basis of the line broadening at half of the maximum intensity of reflection (111). The estimated crystallite size for the AuNCs is 25 nm, which is smaller than the particle size that might consist of a number of crystallites arranged in the same orientation.

### 3.2 Electrochemically Active Surface Area Development by AuNCs

**Figure 2:**
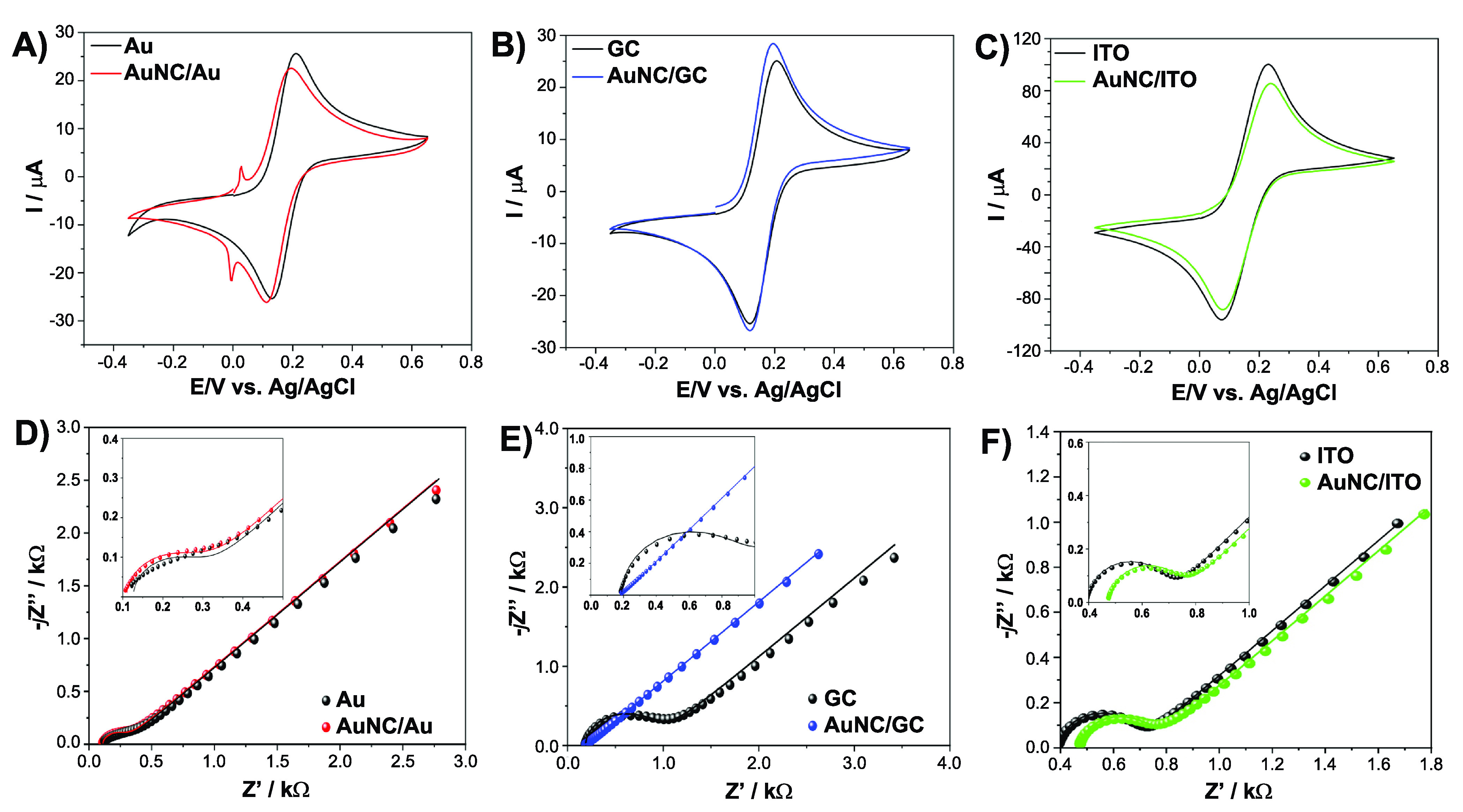
(A–C) CVs and (D–F) EIS data for (A and D) gold, (B and E) GC, and (C and F) ITO electrodes prior to (black) and after deposition of 10 μL of AuNC (colored). Studies in 0.01 M PBS containing 1.0 mM K_3_Fe(CN)_6_.

Deposition of AuNCs on the GC, Au, and ITO electrode substrates was carried out by drop-casting 10 μL of AuNCs suspended in 0.001 M CTAB, leaving the decorated electrodes to dry at room temperature. The deposition resulted in the adsorption of the AuNCs, expectedly resulting in the development of charge transfer kinetics and/or an EASA, to be verified by CV and EIS analyses. The CV results are shown in Figure 2.

Drop-casting AuNCs on the GC surface notably increased the peak redox currents compared with that of the pristine GC electrode (Figure 2B). A similar observation was previously made in the case of decoration with gold nanoparticles and was connected to the increase in electrical conductivity.^56^ Furthermore, the effective increase in the electron transfer kinetics was confirmed by tracking the decrease in anodic–cathodic peak separation (Δ*E*_p_) from 84 to 71 mV after AuNC deposition. A similar feature was observed for Au, but not for ITO, where Δ*E*_p_ remained around 155 mV, suggesting a significantly higher irreversibility of the electron transfer. Furthermore, unlike in the case of the GC substrate, AuNC drop-casting at either ITO or Au decreased the ferrocyanide oxidation/reduction currents, as seen in panels A and C of Figure 2. A repeatable occurrence of small side CV peaks in Figure 2A is probably a result of electrocatalytic hydrogen redox chemistry on gold nanoparticles.^57^ The detailed information is provided in section S3 of the Supporting Information. The electron transfer rate increased at the AuNC/GC electrode due to better electrochemical catalytic behavior. This is a consequence of the high activity of AuNCs.^58^

The ferrocyanide oxidation kinetics made it possible to draw conclusions about the EASA development by AuNC drop-casting. To do this, CV scans were performed at various scan rates (see section S3 of the Supporting Information) to exercise the R–S relationship for the reversible reaction (eq 3).
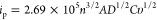
3where *i*_p_ is the peak current in amperes, ν is the scan rate in volts per second, *C* (1 × 10^–6^ mol cm^–3^) is the [Fe(CN)_6_]^3–^ concentration, *D* (6.67 × 10^–6^ cm^2^ s^–1^) is its diffusion coefficient,^59^*n* (=1) is the number of electrons transferred, and *A* is the EASA in square centimeters. On the contrary, heterogeneous rate constant *k*^0^ was estimated from Δ*E*_p_ at a ν of 100 mV s^–1^ using the Nicholson approach^60,61^ (see eq 4).
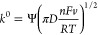
4with Ψ being the dimensionless, empirically determined kinetic parameter. The results are summarized in Table 1.

**Table 1:** Electric Parameters Obtained from EIS Analyses after Fitting with *R*_S_[CPE(*R*_CT_*W*)] EEC

	sample	Au	GC	ITO
*k*^0^ (cm s^–1^)	bare	1.32 × 10^–2^	9.11 × 10^–3^	1.82 × 10^–3^
	AuNC	1.82 × 10^–3^	1.81 × 10^–2^	1.64 × 10^–3^
EASA (cm^2^)	bare	0.72	0.63	0.88
	AuNC	0.92	1.04	0.88
*R*_CT_ (Ω)	bare	219	812	304
	AuNC	322	41	282
*C*_DL_* (μF)	bare	1.25	1.31	1.40
	AuNC	2.31	0.11	1.74
α	bare	0.84	0.89	0.92
	AuNC	0.70	0.82	0.88
*W* (μΩ s^–1/2^)	bare	0.36	0.37	0.91
	AuNC	0.34	0.36	0.86

Importantly, it was noted that the calculated EASA for the GC electrode after AuNC decoration undergoes a tremendous 65% increase, reaching 1.04 cm^2^. A smaller yet notable 27% increase was observed for the Au electrode after decoration, while the AuNC-decorated ITO EASA remained completely unchanged. The obtained results demand answers to questions about the origin of the relevant alteration of the EASA, the mechanism of interaction of AuNCs with different electrode surfaces, and the electron transfer mitigation by CTAB, the surfactant used for AuNC stabilization.

An EIS analysis was also performed for the same batch of samples. The detailed data analysis was performed after fitting the experimental results (Figure 2D–F, points) with an electric equivalent circuit (EEC) (Figure 2D–F, line). The EEC selected, *R*_S_[CPE(*R*_CT_*W*)], was a derivative of the Randles circuit, represented by series resistance *R*_S_ (electrolyte resistance) and charge transfer resistance *R*_CT_, the latter connected in series to the Warburg diffusion impedance (*W*) and in parallel to the constant phase element (CPE), representing the electric double-layer quasi-capacitance (*C*_DL_). The CPE was introduced to consider the frequency dispersion of capacitance upon the occurrence of the electrode electric heterogeneities, introduced upon AuNC decoration in particular, but also present due to other effects, such as the surface roughness and porosity, polycrystallinity, adsorption phenomena, etc.^61^ The CPE’s impedance is defined by eq 5
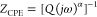
5where *Q* is the quasi-capacitance and CPE exponent α describes the level of surface heterogeneity. The homogeneous surface is represented by the ideal capacitor when α = 1, and the lower the α value, the greater the dispersion of capacitance due to disturbances in the diffusion layer. The surface time constant distribution model was chosen to estimate the effective *C*_DL_* value.^62^ The results of the impedance analysis are summarized in Table 1.

Charge transfer resistance *R*_CT_ of the bare electrode expectedly largely depends on the substrate and, under the studied conditions, is lowest for the ITO and Au electrodes. This parameter manifests changes in the charge transfer pathways. No notable changes were observed upon AuNC decoration on ITO, while for Au, the increase in *R*_CT_ most likely originates from CTAB adsorption at the electrode surface, as discussed below. Furthermore, there are no notable changes observed in this parameter upon AuNC decoration, suggesting that the charge transfer pathways were not significantly affected. A different situation is observed in the case of the GC substrate, where the initially highest *R*_CT_ value decreased by >1 order of magnitude to merely 40 Ω upon AuNC decoration, testifying to the substantial alteration of the electrode transfer kinetics.

An important observation can be made about the capacitance of the electric double layer. The parameter represented by the CPE parameter shows a clear dependence on AuNC decoration. Initially, quasi-capacitance *Q* is similar for all of the studied electrodes with small differences occurring most likely due to different Volta potentials and their influence on the charge accumulation by the electric double layer as well as the surface electric heterogeneities of the studied substrates. The *C*_DL_* parameter notably decreases for the AuNC-decorated GC electrode, yet a similar feature was not observed for the ITO or Au substrate. This is an interesting observation, in particular when combined with the simultaneous decrease in *R*_CT_, suggesting a significantly faster rate at which the charged interface of the electrode is established (time constant τ = *R*_CT_*C*_DL_*), which decreased by >2 orders of magnitude to 4.5 μs. In all cases for the other electrodes, with or without decoration, τ ∼ 0.3–0.7 ms. This effect could possibly be explained by the presence of attraction forces with the electrode substrates and AuNC, easing the electron mediation mechanism.

It should also be noted that the proposed surface functionalization affects the electric heterogeneity of the Au and GC substrates while leaving the ITO nearly unaffected. This observation allows us to hypothesize (i) a very homogeneous distribution of the AuNCs or (ii) a limited interaction of the AuNCs with the ITO surface. At the same time, the results prove a negligible role of the AuNC in the transport of the reagent through the diffusion layer.

**Figure 3:**
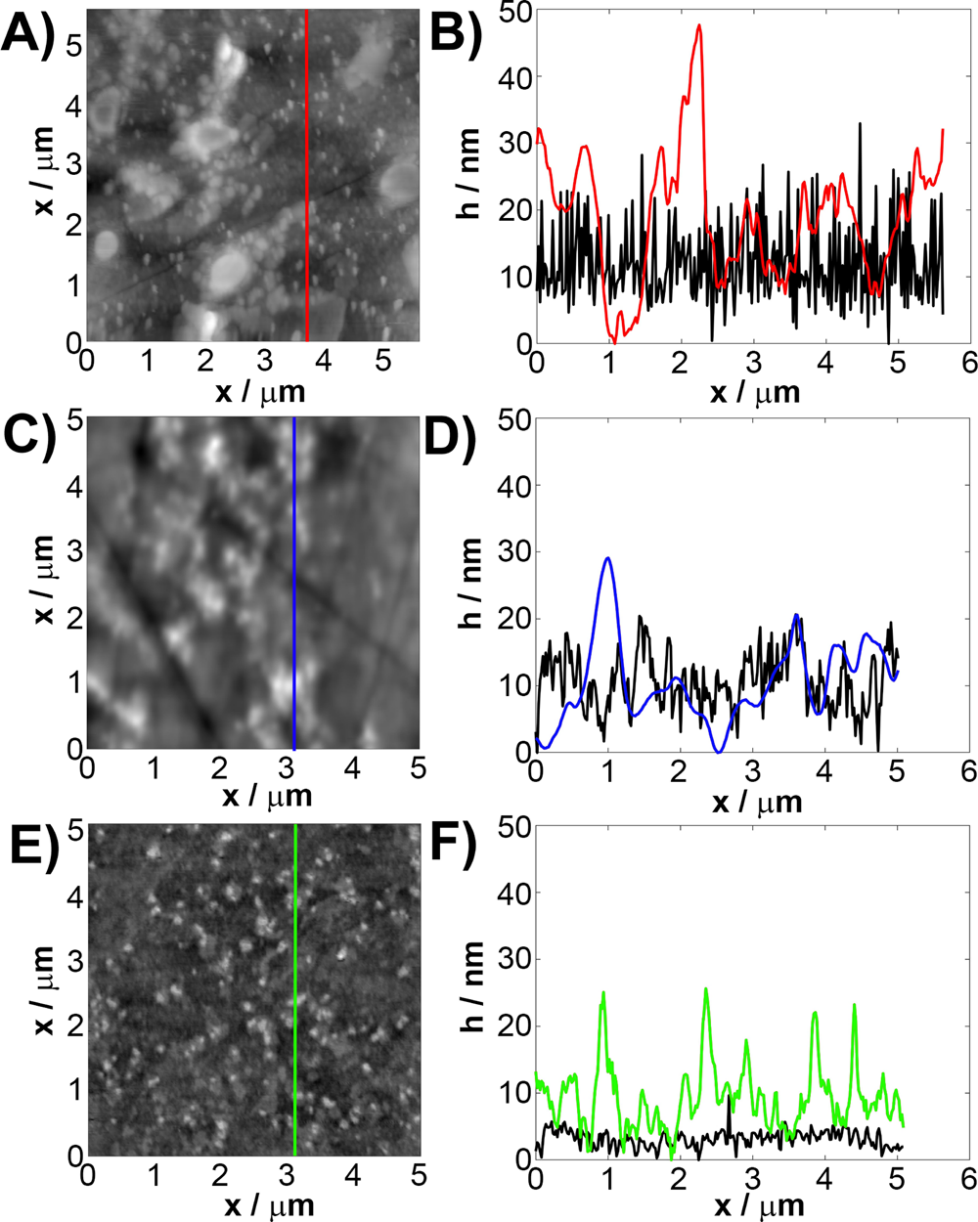
(A, C, and E) AFM semicontact mode topographic images and (B, D, and F) topographic profiles of AuNC-decorated studied samples: (A and B) gold, (C and D) GC, and (E and F) ITO. Black lines represent profiles of bare substrates for comparison.

The results of AFM topographic imaging allow us to discuss the effectiveness of deposition of AuNCs on each of the studied substrates, as shown in Figure 3. These micrographs allow the clear recognition of the modification of the electrode topography upon AuNC solution drop-casting. A follow-up topography profile study determined the average roughness of both substrate electrodes and differentiated the dispersion of nanocubic systems following their agglomeration for each substrate, affecting the homogeneity of the obtained surfaces.

Panels A, C, and E of Figure 3 reveal a complex topography of the AuNC-decorated electrodes, with the reference unmodified samples presented in section S4 of the Supporting Information. At first glance, every surface seems to be enriched with features that can be interpreted as AuNC agglomerates. However, the topography profiles suggest that only in the case of gold and GC electrodes can these features exceed the typical AuNC dimensions of 40 nm. Following this observation, much smaller and more evenly distributed species at the ITO surface (Figure 3E) may have different origins, e.g., CTAB. Section S5 of the Supporting Information describes the AuNC/ITO system after the drop-casting procedure. The amount of deposited AuNCs is negligible when compared to the amount of the GC substrate, while the surface is uniformly covered by the surfactant.

Additionally, a statistical analysis led to roughness parameter *S*_q_ defined by eq 6:
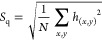
6corresponding to the standard deviation of the height on the AFM images (Figure 3A,C,E and Figure S2) with the number of pixels equal to *N*. A summary of the parameter defined above for images with sizes of 256 pixels × 256 pixels is presented in Table 2. These studies confirm previously obtained information, as the *S*_q_ parameter changes by only 3.15 nm in the case of the ITO sample upon AuNC decoration. On the contrary, the average change in *S*_q_ for the GC electrode reaches 25.32 nm, a result to be expected for the evenly distributed cubic monolayer. An interesting feature observed for the Au substrate shows something of a middle ground, as *S*_q_ was changed by 10.42 nm upon decoration with the nanocubes. Such a response may be explained by weaker interaction between the AuNCs and the substrate in this case, or other phenomena, such as gold self-diffusion.

**Table 2:** AFM-Measured Roughness of the Studied Samples and Analysis of the Statistical Data from SECM Mapping, before and after AuNC Decoration

	sample	Au	GC	ITO
*S*_q_ (nm)	bare	5.08	5.08	1.21
	AuNC	15.60	30.40	4.36
*i*_avg_ (nA)	bare	–9.38	–8.69	–7.65
	AuNC	–5.39	–9.22	–7.79
*i*_Sq_ (pA)	bare	31.2	31.4	45.2
	AuNC	220.1	145.3	36.2
*k*^0^ (cm s^–1^)	bare	3.88 × 10^–2^	2.29 × 10^–2^	1.67 × 10^–2^
	AuNC	6.40 × 10^–4^	4.00 × 10^–2^	1.53 × 10^–2^

### 3.3 Stability and Interactions of Adsorbed AuNC

**Figure 4:**
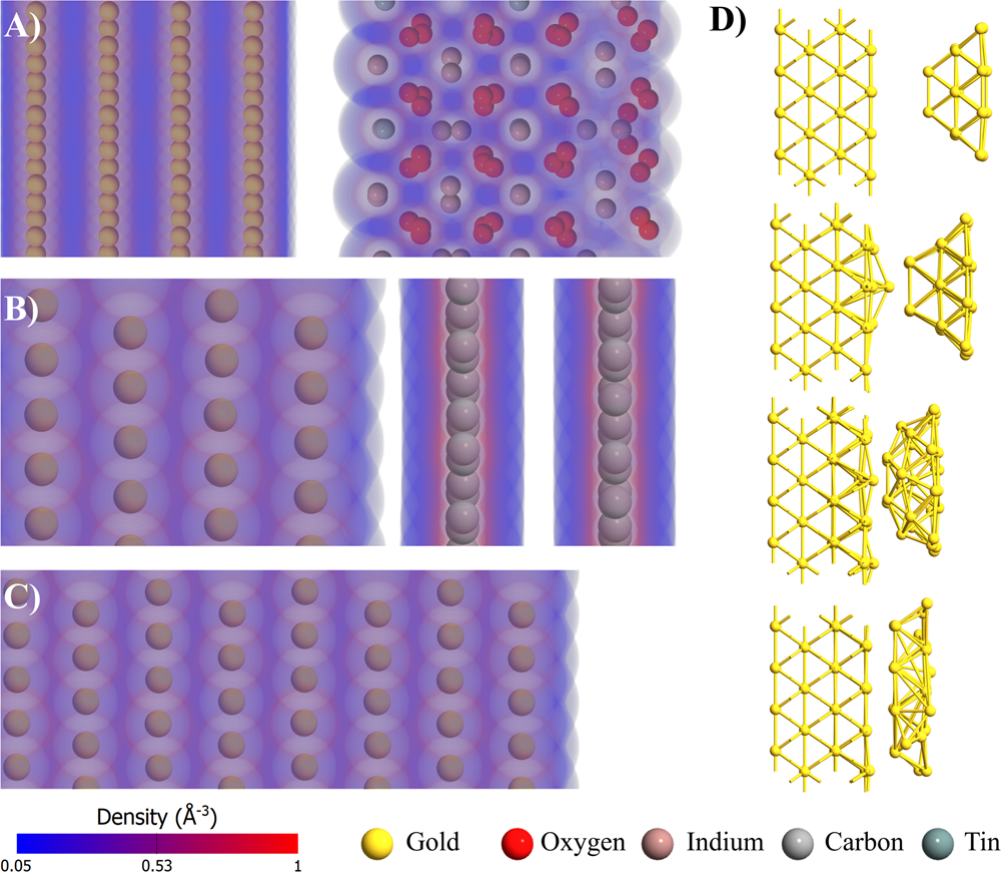
Optimized geometry of interactions between surfaces with electron density maps: (A) Au–ITO, (B) Au–GC, and (C) Au–Au interfaces. (D) Simulation of adsorption of 1 nm gold nanoparticles on flat gold surfaces [the first frame is the as-prepared non-optimized geometry, the second frame after 25 steps, the third frame after 75 steps, and the fourth frame the fully optimized geometry (262 steps)].

To simulate the interactions of the AuNCs with the Au, ITO, and GC surfaces, a series of pairwise interacting Au–ITO, Au–GC, and Au–Au slabs were constructed. Geometry optimization was performed with one of the slabs allowed to move freely (Au) and the other (GC/Au/ITO) fixed in space. Interactions between the surfaces are illustrated in Figure 4A–C, where pictures of optimized geometries along with surface density maps are presented.

In the case of the Au–ITO interface, the optimized distance between the surfaces is equal to 5.4 Å and the electron densities of two surfaces do not overlap. Considering the relatively long distance between these surfaces, electron tunnelling is significantly hampered.^63,64^ Moreover, due to the non-overlapping electron densities, no bonds between surfaces are found, and therefore, the hopping mechanism of charge transfer would not be observed either.^65^ These observations suggest that electrical contact between flat Au–ITO surfaces is not likely to form spontaneously. This corollary can explain the experimentally observed phenomenon that application of AuNCs on the surface of ITO does not result in any change in the EASA with negligible changes in the charge transfer resistance (Table 1) as well as minor disturbances in the AFM-deciphered surface topography (Table 2). However, in the case of the Au–GC interface, the optimized distance is significantly shorter (3.5 Å) and there is a small overlap between the electron density of the Au layer and the carbon layer. The distance is even shorter than the spacing between graphite sheets (4.1 Å) and suggests that charge transfer between Au and GC flat surfaces is likely to occur. This is reflected experimentally in the largest increase in the EASA after application of AuNCs among all three surfaces and the significant decrease in charge transfer resistance.

**Figure 5:**
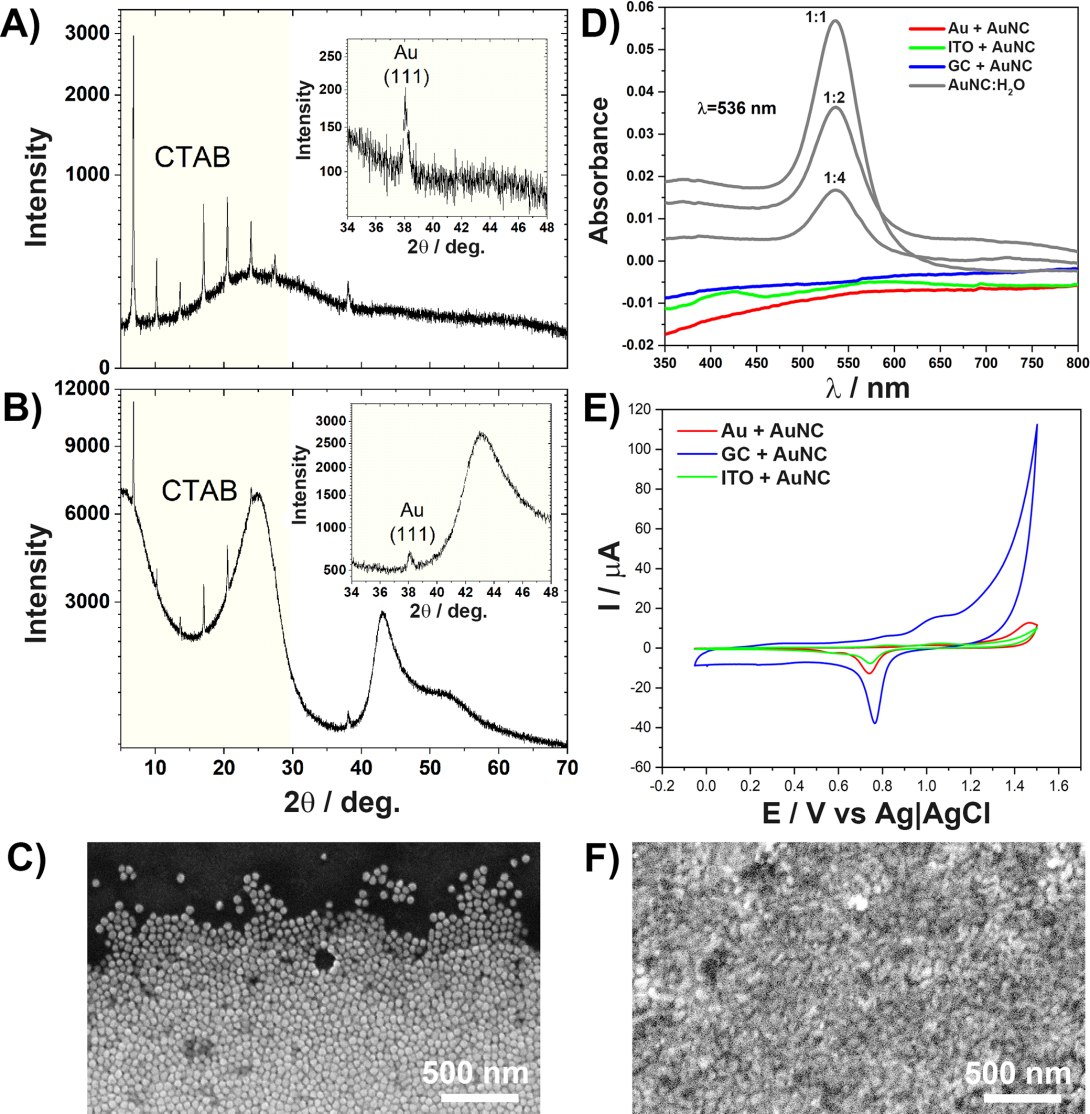
XRD diffraction patterns for (A) AuNC/ITO and (B) AuNC/GC. The inset of panel A shows the XRD pattern of the (111) crystallographic plane. SEM images of (C) AuNC/GC and (F) AuNC/Au surfaces in the area of appearance of the coffee-ring effect. (D) UV–vis absorption spectra recorded after deposition of AuNC on electrodes and in diluted solutions (1:1, 1:2, and 1:4). (E) CVs for electrodes with drop-cast AuNCs in 0.1 M H_2_SO_4_ at a scan rate of 20 mV s^–1^.

Surprising phenomena are observed when Au–Au interactions are considered. The optimized distance between surfaces is equal to 2.4 Å, which is almost on par with the distance between Au layers in the slabs (2.1 Å). In other words, an optimized structure of two parallel Au slabs consisting of four Au layers is almost identical to that with one large Au slab with eight layers. This observation suggests that the presence of Au–Au self-diffusion^66,67^ led to the gluing of the two gold surfaces. To further examine this phenomenon, a geometry optimization was performed with a 1 nm nanoparticle on the flat slab. Several frames from this simulation are depicted in Figure 4D. After 25 optimization steps, the nanoparticle translates toward the flat surface, which extends several gold atoms in the direction of the incoming nanoparticle. Then, frontier layers of the nanoparticle are distorted so that they approach the flat gold surface. Finally, in the last frame of the optimization, the gold nanoparticle is shape-shifted so strongly that two parallel flat surfaces remain. Presumably, this phenomenon is responsible for the AuNC decoration effect on the electrode topography and EASA, when compared to GC as well as the unique surface morphology of Au modified by AuNCs registered by SEM (see Figure 5F).

To confirm the conclusion regarding the significantly altered interaction of the nanocubes with various electrode substrates, four independent physicochemical techniques were proposed, and their results are visualized in Figure 5.

XRD and UV–vis analyses were performed for the AuNC drop-cast (10 μL) electrodes after exposure to the electrolyte to evaluate the strength of adsorption of AuNC at the electrode surface throughout the 30 min exposure to the electrolyte. XRD analyses of AuNCs on the GC and ITO electrode substrates are presented in panels A and B of Figure 5, respectively. The intensity is on the square root scale. For both samples, only one XRD reflection of AuNCs was found on the XRD patterns (see the insets of panels A and B). Comparing the (111) reflections observed at a 2Θ of 38.1°, we found that the Au–GC sample has a doubly intense reflection compared to that of the Au–ITO sample, a feature testifying to differences in AuNC self-assembly on these electrodes and having an impact on the electrochemical properties. The average crystallite size for AuNCs, calculated from the Scherrer formula, is equal to 35 and 30 nm for the Au–GC and Au–ITO samples, respectively. Bragg reflections were observed for the 2Θ range of 5–35° and belong to the CTAB. The relative intensity of CTAB reflections slightly increases for the Au–ITO sample, suggesting stronger adsorption of the CTAB to the ITO than to the GC substrate.

On the contrary, the UV–vis absorption spectra shown in Figure 5D do not indicate the presence of the absorption peak characteristic of AuNCs. The 536 nm absorption peak, characteristic of gold nanocubes^50^ and other colloids,^6^ was recognized only for reference AuNCs in a 0.001 M CTAB solution. The lack of this peak in the case of the decorated electrodes corroborates the presence of the AuNCs on the surface of the electrodes; thus, the employed modification method can be successfully applied for electrochemical measurements.

Moreover, the surfaces of 100 μL of AuNC-decorated electrodes were electrochemically characterized in a 0.1 M H_2_SO_4_ solution. As shown in Figure 5E, for all of the CVs characteristic of Au^0^|Au^3+^, oxidation and reduction peaks are observed. The shape of the voltammograms is characteristic of the presence of AuNPs, which is different from the bare Au electrode in an acidic solution.^68,69^ The oxidation peak is recorded at 1.04 V for AuNCs/GC and AuNCs/ITO, whereas for AuNCs/Au, it is shifted to 1.46 V. The irreversible Au_2_O_3_ reduction occurs at 0.76 V (AuNCs/GC) or 0.74 V (AuNCs/ITO and AuNCs/Au).^70−72^ Tracking the area under the cathodic peak makes it possible to analyze the total charge that has flowed during Au_2_O_3_ electroreduction at the AuNC surface. This phenomenon is attributed to electrocatalytic active sites.^73^ The smallest amount of electrocatalytic active sites is present on AuNC/ITO electrode (2 times less than AuNC/Au), and the largest amount on AuNC/GC (4.5 times more than AuNC/Au), which also contributes to the presence of AuNCs on the electrode surface.

We were unable to find any AuNCs drop-cast at the Au surface by SEM observations, even when the analysis was performed within 5 min of the decoration. Thus, we greatly increased the volume of cast AuNCs and focused on the outer ring of the droplet, where the concentration is supposed to be the highest on the basis of the appearance of the coffee-ring effect. Comparative analyses of the coffee-ring effect formed by the AuNC at GC and Au are given in panels C and F of Figure 5, respectively. Rapid Au–Au self-diffusion has rapidly led to decay in the nanocubic structure, only slightly disturbing the observed surface topography.

### 3.4 Influence of AuNC on the Distribution of the Diffusion Field

**Figure 6:**
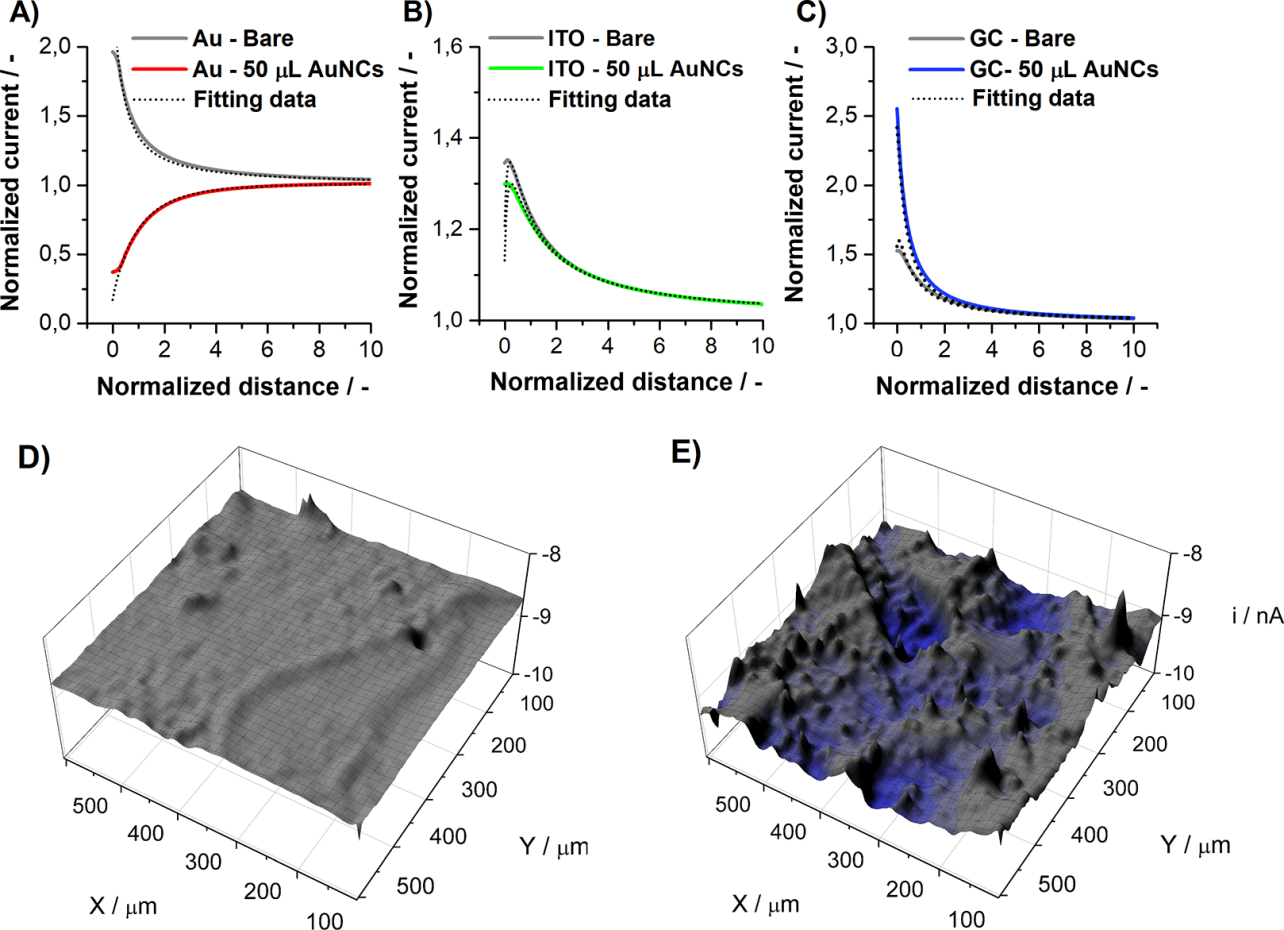
Approach curves for samples before and after drop-casting of AuNCs on different substrates: (A) Au, (B) ITO, and (C) GC. SECM maps of the GC surface (D) before and (E) after AuNC drop-casting, imaged at a working distance of 5 μm. The redox probe was Fe(CN)_6_^3–^.

The approach curves were registered at the beginning of each SECM measurement to determine the position of each sample. These characteristics provide valuable information about the kinetics of the electrochemical reaction occurring at the surface of the electrode under investigation.^74^ Standardized data obtained during the approach are shown in Figure 6A–C. The normalized current was plotted versus the normalized distance, and more detailed information is presented in section S6 of the Supporting Information. After a successful approach, surface imaging was performed at a working distance of 5 μm. Panels D and E of Figure 6 present the SECM mapping of the GC surface before and after AuNC drop-casting, respectively. The SECM maps of the remaining electrodes are shown in Figure S5.

For all electrode materials prior to AuNC decoration, the approach curves represent positive feedback, meaning spontaneous regeneration of the mediator occurring at each studied surface.^75^ By fitting the approach curve for each material, we obtained the Fe(CN)_6_^3–^ reduction rate constant *k*^0^ [at −0.2 V vs Ag/AgCl(gel)]. The *k*^0^ values are listed in Table 2, and the procedure is explained in detail in section S6.2 of the Supporting Information. The *k*^0^ for the bare substrates increases sequentially: ITO < GC < Au (likewise calculated from the CVs in Table 1). Also, similar to Table 1, the *k*^0^ for Au decreases after AuNC deposition. Here, the negative feedback of the approach curve reveals that regeneration of the mediator has been blocked. There is only a slight change in the rate constant in the case of AuNCs/ITO, while an increase in *k*^0^ was observed after GC decoration. All observations are in good correlation with the data obtained using the Nicholson approach.

Importantly, for the AuNCs/GC, the current registered at the tip of the microelectrode decreased upon reaching the touching point and a second, identical approach curve could not be obtained without additional polishing of the microelectrode. This behavior was explained by desorption of CTAB from the AuNC/GC surface and its attachment to the active part of the microelectrode, suggesting greater energy for adsorption of CTAB to Au than to GC, as explained below. The SEM images of the microelectrode taken after the SECM experiment are shown in Figure S4. Moreover, the approach curve toward the AuNCs/GC surface is the only one without a clear touching point, as current overflow occurred. This can be explained by exchanging electrons directly between the substrate surface and the microelectrode tip by the tunneling effect.^76^

Current distortion *i*_Sq_, describing profile height deviations, was determined by analyzing the SECM maps. It is defined in the same way as AFM roughness *S*_q_, although computed from whole area of the sample. In SECM studies, *i*_Sq_ is connected to the surface topography and local electrode kinetics; thus, higher values are expected after AuNC deposition. All of the data from the analysis were obtained for all surfaces and are listed in Table 2.

The highest average current value *i*_avg_ is observed for the bare Au electrode and decreases significantly after AuNC drop-casting. Such behavior corresponds well with the data from the approach curves and DFT simulations and can be assigned to the stronger interactions between the Au and CTAB molecules, hindering the electron transfer through the interphase. In contrast, *i*_avg_ increases for the GC after AuNC deposition, which may be explained by the strong interactions of the AuNCs with the GC surface and weak interaction between the GC and CTAB. Among all of the studied systems, the ITO appears to be affected the least by the presence of AuNC.

As one can see in Table 2, the heterogeneity of the charge transfer within the diffusion field increases upon drop-casting for GC and Au. This observation corroborates perfectly what we already know about the AuNC self-assembly mechanism on these substrates. For the Au substrate, as *i*_avg_ decreases upon AuNC decoration, the increase in *i*_Sq_ is most likely connected to locally altered aggregation of CTAB molecules that are hindering electron transfer. This is supported by the topography changes seen in Figure 3. On the contrary, such a situation does not occur for the GC, for which *i*_Sq_ is directly connected to the increase in EASA and improvement of the charge transfer kinetics. The fact that the roughness parameter does not change for the ITO surface could be caused by a weak attraction of ITO to both AuNCs and (unlike the Au substrate) CTAB, providing a negligible change in the electrochemical response.

**Figure 7:**
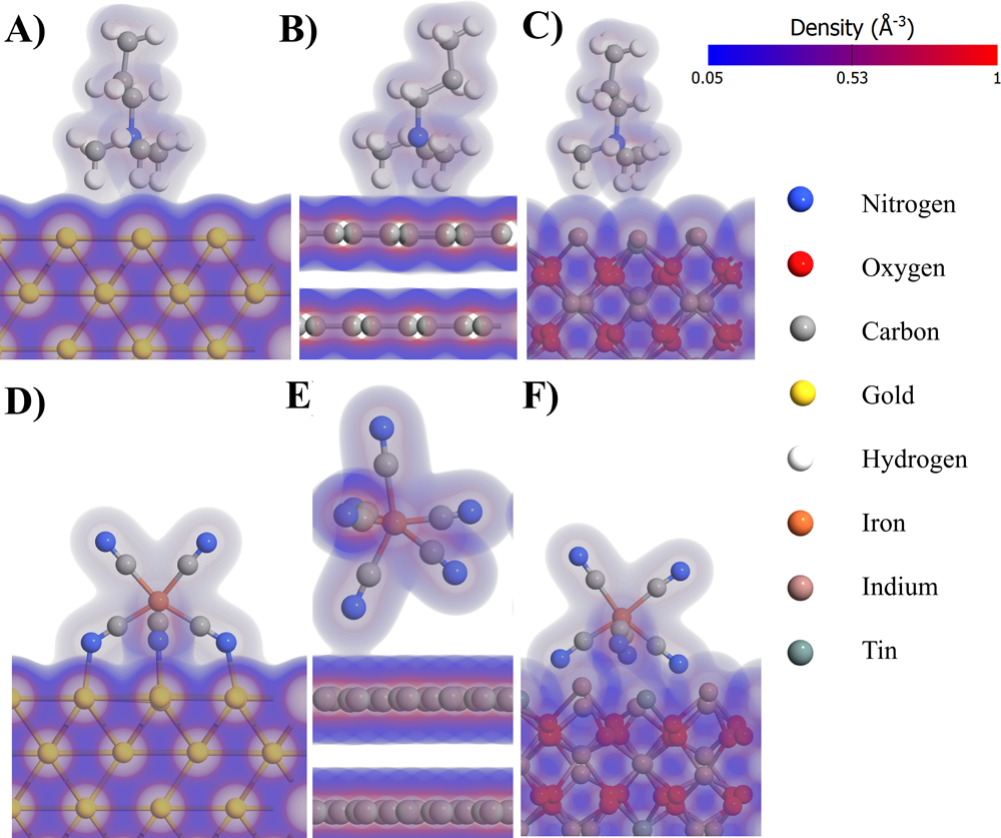
Electron density maps of adsorption of (A–C) CTAB and (D–F) ferrocyanide on different surfaces: (A and D) Au, (B and E) GC, and (C and F) ITO.

To support the electrochemical results further, adsorption of the CTAB surfactant and (FeCN_6_)^4–^ on Au, GC, and ITO surfaces was simulated (Figure 7). To the best of our knowledge, DFT analysis of the adsorption of ferrocyanides on those surfaces is reported for the first time. For computational reasons, the alkyl chain of the CTAB was shortened so that the studied molecule is *N*-trimethyl isopropylamine. The effects of counterions were neglected. CTAB adsorbs on all three surfaces in a similar geometry with three electrostatic bonds and a small degree of overlapping electron density. In the case of Au, the C–H–Au bond lengths vary in the range of 2.53–2.64 Å, GC C–H–C bond lengths in the range of 2.41–2.54 Å, and ITO bond lengths in the range of 2.87–3.06 Å. Negative values of the Gibbs energy for CTAB adsorption strongly suggest that those processes are thermodynamically favorable for all three surfaces. However, the lowest energetic drive is observed for GC and is equal to −0.49 eV (Table 3). This fact might explain why the intensities of the CTAB signals observed on XRD are higher for the Au substrate than for the GC substrate.

**Table 3:** Adsorption Energies (in electronvolts) of CTAB and Ferrocyanides on Different Surfaces

	Au	GC	ITO
CTAB	–2.28	–0.49	–2.15
Fe(CN)_6_^4–^	–7.74	–4.36	–7.77
Fe(CN)_6_^4–^ in the presence of adsorbed CTAB	–5.46	–3.87	–5.63
Fe(CN)_6_^4–^ disturbance energy due to the presence of CTAB	2.28	0.49	2.15

Adsorption of (FeCN_6_)^4–^ on Au occurs via formation of four surface bonds between Au atoms and nitrogen atoms from cyanide groups with lengths from 2.13 to 2.16 Å. Considering their relatively short length and high degree of electron density overlap, the nature of those N–Au bonds is highly covalent, in contrast to the case for CTAB. On the contrary, the adsorption of (FeCN_6_)^4–^ on GC is very weak and monodentate. A 2.97 Å N–C bond is formed with minimal overlap of electron density, suggesting an electrostatic character of this interaction. This observation explains the fact that before the modification with AuNCs, glassy carbon exhibits the highest charge transfer resistance (see Table 1). The strongest adsorption of (FeCN_6_)^4–^ occurs on the surface of ITO. The geometry of the formed covalent/coordination bonds is similar to that in the case of gold; however, the overlap of electron densities is significantly larger. Surface bonds are created between cyanide nitrogen atoms and both indium and tin on the ITO side; in particular, the In–N bond lengths vary in the range of 2.11–2.28 Å, and the Sn–N bond lengths are equal to 2.55 Å. Similarly, as in the case of CTAB, the free energies of (FeCN_6_)^4–^ adsorption are negative for all three surfaces and the thermodynamic drive is the weakest for GC, while for ITO and Au, its strength is comparable (approximately −7.7 eV). Considering that the adsorption of ferrocyanide ions is the first step in the mechanism of electrode reactions,^75^ the adsorption Gibbs free energy can be used as an indirect measure relating the experimentally calculated electrochemical properties to the ab initio simulations.

The disturbance energy is defined as the difference between the ferrocyanide adsorption energy on the pristine surface and the adsorption energy on the CTAB-occupied surface by eqs 1 and 2 and is equal to the CTAB adsorption energy with a negative sign. If the surface is already occupied with CTAB, the adsorption free energies are still negative for all surfaces but are shifted to higher values (Table 3). However, the magnitude of this shift (disturbance energy) for Au and ITO is equal to ∼2.2 eV, while that of glassy carbon is shifted by only 0.5 eV. In other words, the surface at which CTAB disturbs ferrocyanide adsorption the least is GC. This result stands in perfect agreement with the voltammograms and EIS spectra in Figure 2. After application of AuNCs on GC, the electrochemical activity increases toward the ferrocyanide redox pair and is the highest among all three of the surfaces because the potentially blocking effect of CTAB is minimal.

**Figure 8:**
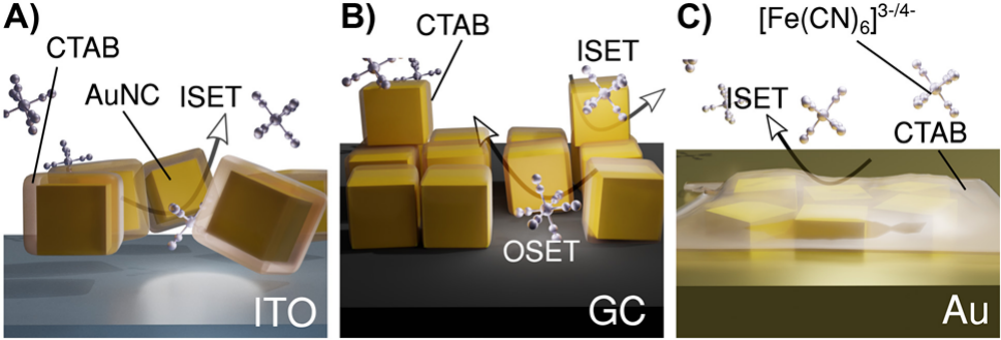
Schematic illustration of the self-assembly of AuNCs at (A) ITO, (B) GC, and (C) Au surfaces and modulation of the charge transfer mechanism by [Fe(CN)_6_]^3–^ and [Fe(CN)_6_]^4–^.

Moreover, reasoning based on the adsorption geometries suggests that the charge transfer between the ferrocyanide and electrode observed in the experiments occurs via the ISET (inner sphere electron transfer) mechanism on the Au and ITO but via OSET (outer sphere electron transfer) on the GC.^77^ Therefore, the activation energy of the electron transfer on the GC is expected to be the highest,^78^ which is reflected in the electrochemical results by the highest overpotentials and slowest charge transfer kinetics. On the contrary, the height of the electron transfer energy barrier on the GC surface should be less prone to change with distortion of the coordination environment, which is typical for OSET reactions.^75^ In other words, longer distances of electron tunnelling from the d orbitals of ferrocyanide through the solvent layers to the quasi-continuous energy levels of the GC electrode are more likely than to the ITO and Au electrodes. As a result, in the SECM experiment, a reduction signal from both the AuNC and the substrate is observed only for the GC template. Notably, the enhancement of the electrochemical performance by AuNC/GC was previously discussed to be solely contributed by the good electrical conductivity of the electrode surface.^56^ Our findings also explain the increasing Fe(CN)_6_^3–^ reduction rate constant *k*^0^, which is noted only in the case of AuNC/GC. Upon decoration with AuNCs, the ISET reaction of gold is competitive with the OSET reaction of GC.^77^ This situation is schematically visualized in Figure 8.

The scheme in Figure 8 illustrates different mechanisms of interaction of AuNC and electrode substrates with CTAB. As revealed with the electrochemical studies and DFT simulations, only the GC among the studied surfaces creates optimal conditions for AuNC self-assembly by developing agglomerates and enhancing both the electrocatalytic activity and the electrochemically active surface area of the electrode (Figure 8B). Moreover, a relatively low energy of adsorption of CTAB to the GC substrate, as seen in the XPS analysis, is not a critical aspect with respect to the charge transfer by the redox probe.

In the case of the AuNC/ITO interface (Figure 8A), the electron densities of the two surfaces do not overlap; thus, the electrical contact is not likely to form spontaneously by by the hopping or tunnelling mechanism.^63^ This is further reflected by the negligible presence of physisorbed AuNC at the ITO surface, as revealed by the XRD, UV–vis, AFM, and CV studies. Additionally, the CTAB energy of adsorption is slightly stronger toward Au than toward ITO, indicating a stronger propensity for adsorption.^50^ Overall, the AuNC/Au surface remains intact after decoration and neither EASA nor the charge transfer kinetics is affected. Finally, due to self-diffusion of AuNC into the Au substrate, both surfaces are spontaneously gluing (Figure 8C), which has a minor effect on the topography and EASA development in comparison to the other substrates. The AuNC/Au surface is the most electrochemically heterogeneous among all of the studied systems, which is presumably due to the appearance of non-uniform CTAB adsorbed layers in the AuNC agglomerate after-effects.^50^ Therefore, due to CTAB adsorption, the Fe(CN)_6_^3–^ reduction rate constant *k*^0^, estimated from the CV and SECM studies, decreases by >1 order of magnitude.

Notably, one should also consider that the geometric factor arising from various interactions of AuNCs, but also (FeCN_6_)^4–^ and CTAB, with the studied surfaces may have an influence on the R–S equation (eq 3), even for the reversible processes. On the basis of the theoretical analysis of the Laplace space, it has been noticed that there may be a relationship in which *i*_p_ ∝ *v*^*p*^, where *p* = (*D*_H_ – 1)/2 and *D*_H_ is the Hausdorff dimension of the analyzed surface.^79^ The statistical quality of the fit of the relationship given in section S3 of the Supporting Information is much better in the case of AuNC cast at the ITO and Au substrates when the scan rate ν exponent is different from 0.5. In the case of AuNC/GC, we could not find a better fit than 0.5, i.e., two dimensions. These data are summarized in section S7 of the Supporting Information. This interesting finding suggests that the improvement of the R–S relationship is obtained by decreasing the dimensionality for the AuNC/ITO and AuNC/Au systems rather than by increasing the EASA. Moreover, the observed trend contradicts the changes in the AFM roughness (Table 2). It turns out that improving the fit by changing the Hausdorff dimension should be explained by modification of the electrode kinetics through increasing the irreversibility of the redox process.

The effects of the electrochemical heterogeneity and the geometry of the diffusion fields are intertwined and in principle cannot be deconvoluted, because both are spatially dependent and the surface morphology determines both effects.^80,81^ CTAB decreases the local kinetic constant because the alignment of energy levels for electron transfer is altered so that adsorption of the ferrocyanide is energetically more demanding, as shown in the DFT results. Locally, kinetic and diffusive effects can act synergistically or antagonistically depending on the spatial distribution of the CTAB deposits.

## 4 Conclusions

In summary, we have demonstrated that the molecular mechanism of assembly of gold nanocubes (AuNCs) at commonly utilized electrode surfaces is different and strongly affects the resultant electrocatalytic effect, and thus eventually the electrode selection. The most valuable findings drawn from this study are summarized here.

- The synthesized AuNCs (38 ± 2 nm), the dominating patterns being the (111) and (200) crystallographic planes, self-assembled at the GC electrode as confirmed by SEM and AFM analyses (*S*_q_ = 30.04 nm).
- The development of the electrochemically active surface area after AuNC decoration showed a tremendous 65% increase for the GC electrode and a less notable 27% increase for the Au electrode, while that of ITO remained unchanged.
- DFT simulations manifested that the enhanced electrocatalytic effect is driven by specific adsorption of ferrocyanide and CTAB at the electrodes, revealing the ISET mechanism on Au and ITO and the OSET on GC.
- The AuNC decoration at GC revealed improved electron transfer kinetics, with the highest heterogeneous rate constant among all studied systems, reaching 1.81 × 10^–2^ and 4.00 × 10^–2^ cm s^–1^ in CV and SECM studies, respectively.
- The accelerated electrocatalytic effect at GC employed heterogeneous diffusion field confinement as well as the lowest electron transfer mitigation by CTAB, the surfactant used for AuNC stabilization.
- A similar electrocatalytic effect was not observed for the ITO or Au substrates. For the latter, the electron transfer rate was confined due to Au–Au self-diffusion and the presence of CTAB.
- The XRD, UV–vis, AFM, and CV studies revealed diminished AuNC physisorption at the ITO substrate due to strong repulsive forces, explaining the immutability of both EASA and charge transfer.
- The CTAB may play an important role in increasing the electrode’s electric heterogeneity, in particular for the AuNC–Au system. The effect results from the energy of adsorption of CTAB to the studied substrate and the influence of AuNC on the surface topography.

There are papers in the literature on the use of gold nanoparticles at electrodes in biosensing, electrocatalysis, and other electrochemical research. Nevertheless, no explicit evaluation of the impact of gold nanoparticles on the electrode performance or the electrocatalytic mechanism that aids in understanding and improving the surface kinetics has been published so far. We have collectively elaborated and revealed the particular gold particle framework effect, paving the way to assessing the efficacy of electron transfer behavior at the nanoscale considering the molecular interactions in the tailored electroanalytical and electrocatalytical application.

## Supporting Information Available

Additional experimental details, including CV, SECM, and XPS analysis for AuNC drop-casted at each of the investigated surfaces (GC, ITO, and Au), and AFM substrate topography maps (PDF)
