# Supplementary material for: Deciphering the Molecular Mechanism of Substrate-Induced Assembly of Gold Nanocube Arrays toward an Accelerated Electrocatalytic Effect Employing Heterogeneous Diffusion Field Confinement
Source: Langmuir. 2022 Jul 27;38(31):9597–610. doi: 10.1021/acs.langmuir.2c01001 (PMC9367014; doi:10.1021/acs.langmuir.2c01001)
Supplement: Supplementary file 1 — la2c01001_si_001.pdf [file la2c01001_si_001.pdf]

Supplementary Information file

For

Deciphering the molecular mechanism of substrate-induced assembling of gold nanocube arrays toward accelerated electrocatalytic effect employing heterogeneous diffusion fields confinement

Pawel Niedzialkowski[1], Adrian Koterwa[1], Adrian Olejnik[2,3], Artur Zielinski[4], Karolina Gornicka[5], Mateusz Brodowski[5], Robert Bogdanowicz[2] and Jacek Ryl[5,*]

[1]Department of Analytic Chemistry, University of Gdańsk, Wita Stwosza 63, 80-952 Gdańsk, Poland
[2]Department of Metrology and Optoelectronics, Gdańsk University of Technology, Narutowicza 11/12, 80-233 Gdańsk, Poland
[3]Centre for Plasma and Laser Engineering, The Szewalski Institute of Fluid-Flow Machinery, Polish Academy of Sciences, Fiszera 14, Gdańsk 80-231, Poland
[4]Department of Electrochemistry, Corrosion and Materials Engineering, Gdańsk University of Technology, Narutowicza 11/12, 80-233 Gdańsk, Poland
[5]Institute of Nanotechnology and Materials Engineering and Advanced Materials Center, Gdańsk University of Technology, Narutowicza 11/12, 80-233 Gdańsk, Poland

* corresponding author (J.R): jacek.ryl@pg.edu.pl

S1. The schematic representation of the location for SECM and AFM measurements

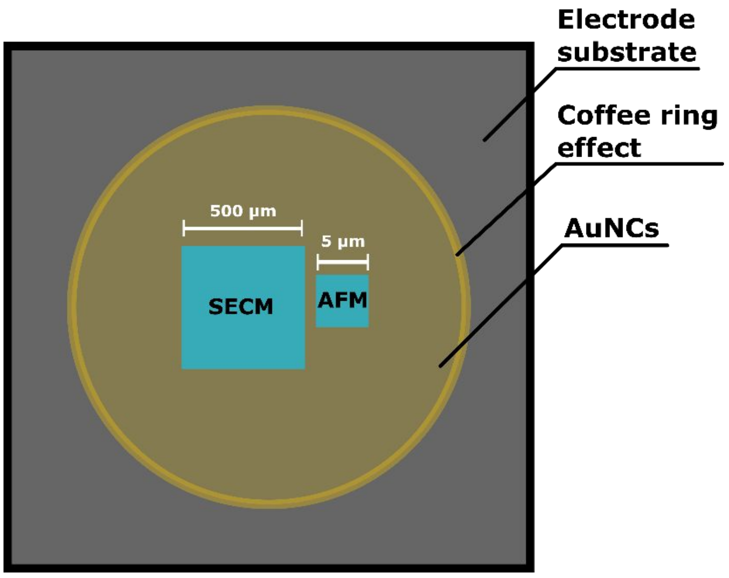

S2. High-resolution *Au 4f$_{7/2}$* XPS results for AuNC drop-casted at ITO and Au substrates

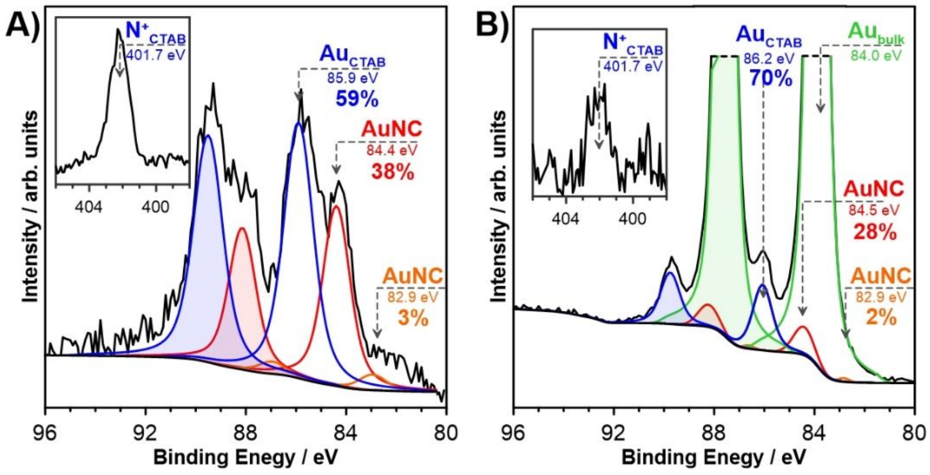

Fig S1 – XPS analysis in *Au 4f* core-level energy range for AuNCs decorated on A) ITO and B) Au substrates. The *N 1s* spectrum in the inset.

The deconvoluted data reveal the presence of the same spectral components as in the case of decorated GC electrodes. On the other hand, the share of each oxidation state within the AuNCs was different, suggesting a much higher amount of Au(III) CTAB-complexed species for both ITO and Au (59% and 70% total [Au], respectively), compared with GC substrate (13%). The presence of CTAB at the electrode surface was confirmed by *N 1s* spectra, where a single peak at approx. 401.7 eV originates from N^{+}_{CTAB} species.

The Au substrate peaks Au^0 bulk Au $4f_{7/2}$ signal at 84.0 eV. Its contribution was not accounted for when calculating the shares of species contribution to AuNC chemistry. However, it should be

considered that its presence may underestimate the AuNC peak at 84.5 eV and hinder the actual Au_{CTAB} ratio. A smaller share of Au(III) species complexed by CTAB is hinted at by the low intensity of the *N 1s* signal for Au substrate.

S3. The cyclic voltammetry studies of the AuNC decorated Au, ITO, and GC electrode kinetics.

Results of cyclic voltammograms of bare substrates and electrodes after drop-casting of 10 µL of AuNCs in 0.01 M PBS pH 7.4 containing 1 mM $K_3[Fe(CN)_6]$ and $K_4[Fe(CN)_6]$ at various scan rates: 0.5, 1, 2, 5, 10, 20, 50, 100, 200, 500 mV s^{-1} and the R-S relationship for $[Fe(CN)_6]^{3-/4-}$ oxidation/reduction peaks vs $\sqrt{\nu}$, obtained for different electrode configurations.

S3.1. GC electrode

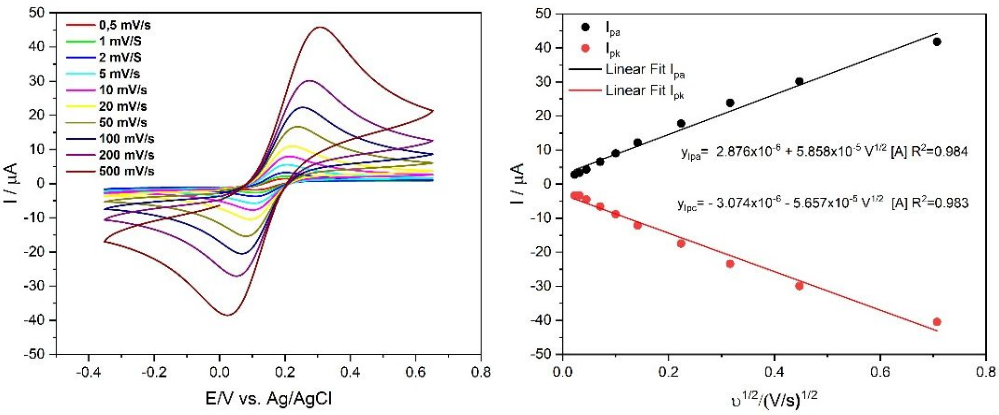

S3.2. AuNCs/GC system

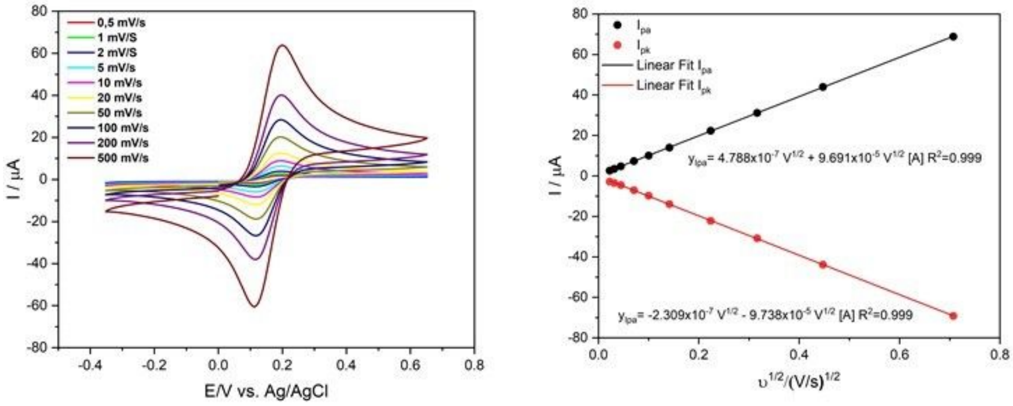

S3.3. Au electrode

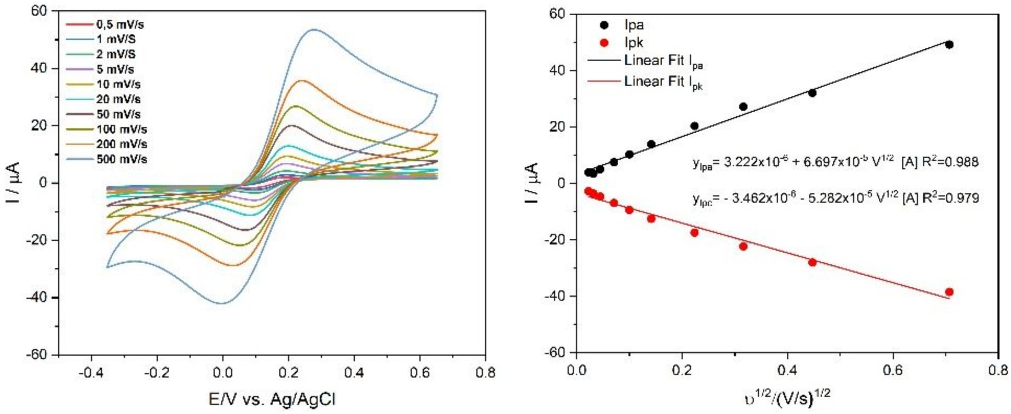

S3.4. AuNCs/Au system

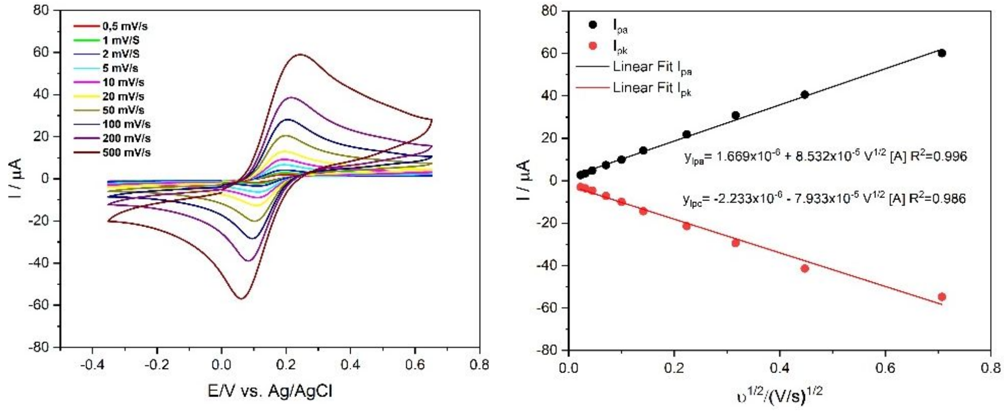

S3.5. ITO electrode

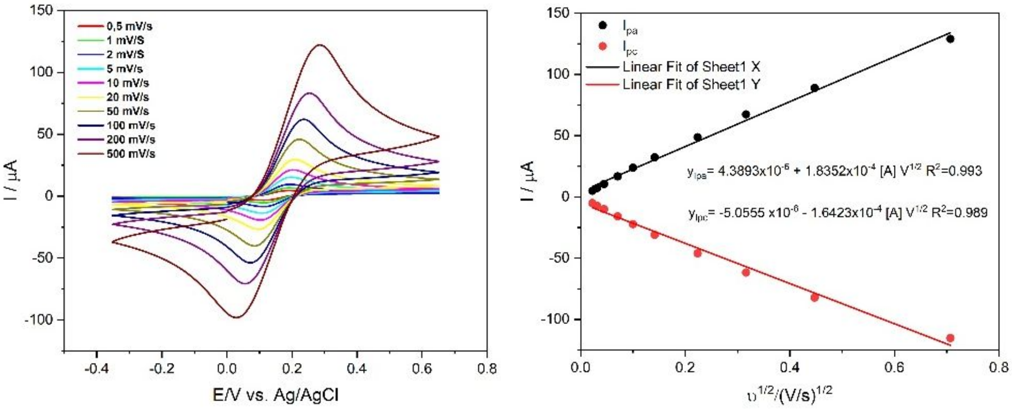

S3.6. AuNCs/ITO system

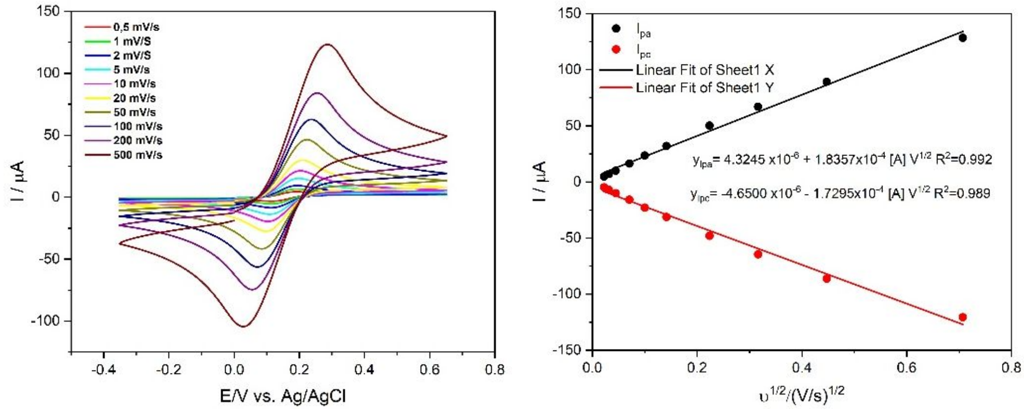

Table S1 - Characteristic CV parameters obtained for each studied electrode configuration at the 100 mV s^{-1} scan rate.

	ΔE_p / V		$i_{A\ 100\ mV/s}$ / µA		$i_{C\ 100\ mV/s}$ / µA	
	bare	AuNC	bare	AuNC	bare	AuNC
Au	81	71	25.8	21.1	-25.0	-26.2
GC	84	71	25.7	28.4	-26.0	-28.4
ITO	151	155	60.5	50.7	-57.6	-53.1

S4. The topography AFM mapping of reference electrode surfaces

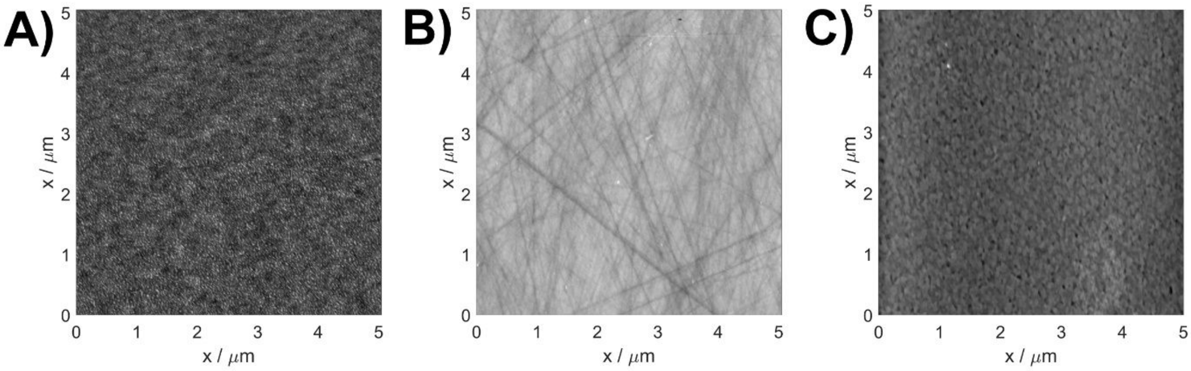

Fig S2 – AFM topographic images of reference samples: A) Au; B) GC; C) ITO

S5. The SEM micrograph of the ITO surface after AuNC drop-casting

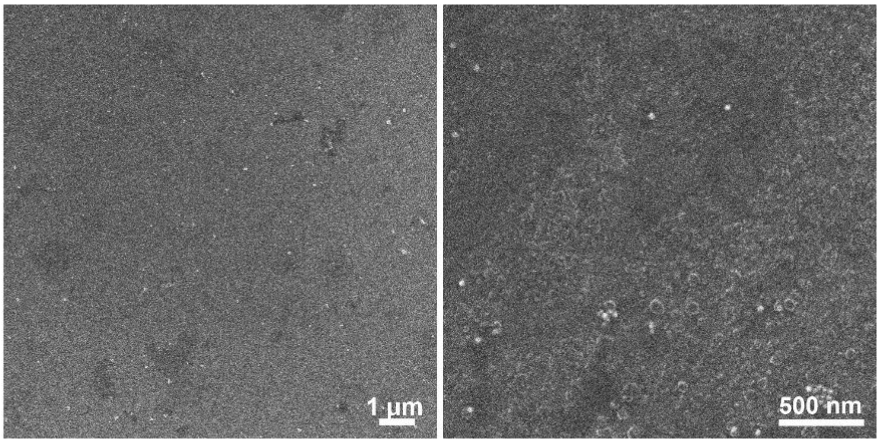

S6. Scanning electrochemical microscopy (SECM) measurements

S6.1. Cyclic voltammetry at the gold microelectrode before SECM measurements

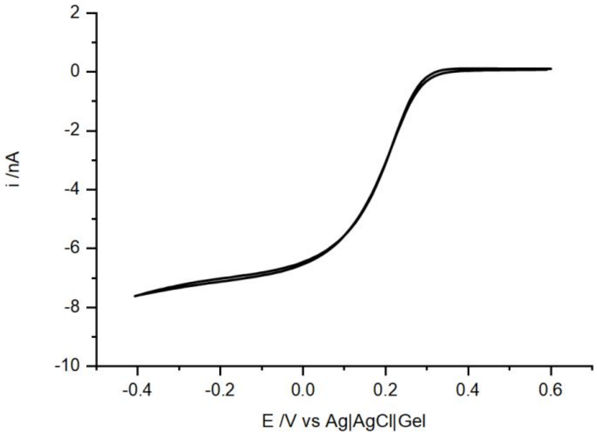

Fig. S3 – The CV registered for gold microelectrode in deoxygenated 5 mM $K_3[Fe(CN)_6]$ in PBS, scan rate 25 mV s^{-1}.

S6.2. Fitting of steady-state SECM approach curves

The most basic parameters that need to be determined for calculations presented in this section are the radius of the microelectrode active part (r_T) and the radius of the insulating part (glass) (r_{glass}). The r_T and r_{glass} have been obtained by SEM imagining of the microelectrode. The SEM images of the microelectrode taken after SECM scan are presented in **Fig. S4.** Next, the RG parameter has been computed as: RG = r_{glass} / r_T.

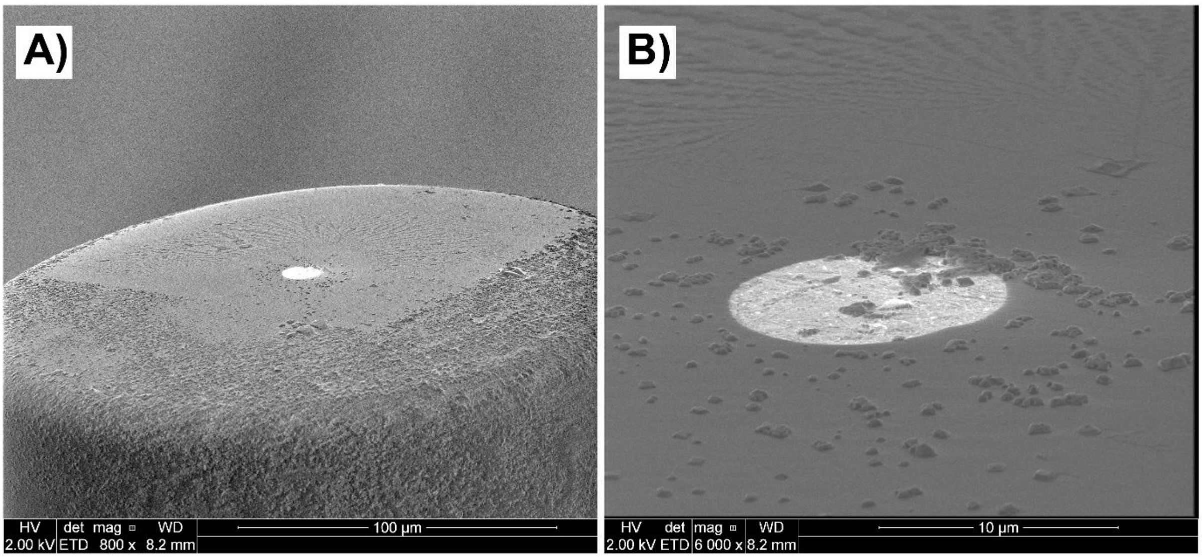

Fig. S4 - SEM images for A) microelectrode embedded in glass, B) microelectrode active part

The approach curves have been normalized according to the following formula (eqs S1, S2):

$$I_T = \frac{i_T}{i_{T,\infty}} \qquad \text{(S1)}$$

$$L = \frac{l}{r_T} \qquad \text{(S2)}$$

The normalized current (I_T) is the proportion between tip current (i_T) and tip current at an infinite distance from the substrate ($i_{T,\infty}$). The normalized distance is expressed as the ratio of the electrode's relative position to the active microelectrode part.

To calculate the theoretical, I_t value for each experiment the approximation of Cornut and Lefrou[S1] was used. The main equations utilized are briefly presented below (eqs (S3-S8)), and a full description can be found in the original work [S1].

$$I_T(L, \kappa, RG) = I_T{}^{cond}\left(L + \frac{1}{\kappa}, RG\right) + \frac{I_T{}^{ins}(L,RG) - 1}{(1 + 2.47RG^{0.31}L\,\kappa)(1 + L^{0.006RG + 0.113}\,\kappa^{-0.0236RG + 0.91})} \qquad \text{(S3)}$$

$$I_T{}^{cond}\left(L+\frac{1}{\kappa},RG\right)= \propto(RG)+\frac{1}{2\beta(RG)\xi\left(L+\frac{1}{\kappa}\right)}+\left(1-\propto(RG)-\frac{1}{2\beta(RG)}\right)\xi\left(L+\frac{1}{\kappa}\right) \quad \text{(S4)}$$

$$I_T{}^{ins}(L,RG)=\frac{\frac{2.08}{RG^{0.358}}\left(L-\frac{0.145}{RG}\right)+1.585}{\frac{2.08}{RG^{0.358}}(L+0.0023RG)+1.57+\frac{\ln RG}{L}+\frac{2}{\pi RG}\ln\left(1+\frac{\pi RG}{2L}\right)} \quad \text{(S5)}$$

$$\alpha(RG)= \ln 2+\ln 2\left(1-\frac{2}{\pi}arccos\left(\frac{1}{RG}\right)\right)-\ln 2\left(1-\left(\frac{2}{\pi}arccos\left(\frac{1}{RG}\right)\right)^2\right) \quad \text{(S6)}$$

$$\beta(RG)= 1+0.639\left(1-\frac{2}{\pi}arccos\left(\frac{1}{RG}\right)\right)-0.186\left(1-\left(\frac{2}{\pi}arccos\left(\frac{1}{RG}\right)\right)^2\right) \quad \text{(S7)}$$

$$\xi\left(L+\frac{1}{\kappa}\right)=\frac{2}{\pi}arctan\left(L+\frac{1}{\kappa}\right) \quad \text{(S8)}$$

Fitting data obtained from experiments allowed us to estimate the normalized constant rate (κ) from the SECM approach curves. which later allowed us to calculate the heterogeneous rate constant (k^0) of first-order reaction according to eq. (3):

$$\kappa=\frac{k^0 r_T}{D} \quad \text{(S9)}$$

As can be seen in eq. (S3) the parameters r_T and D are constant for all measurements presented in this specific work. The κ values for each studied setup were summarized in **Table S2.**

Table S2 – κ values estimated from SECM the approach curves.

**Parameter**	**Sample**	**Au**	**GC**	**ITO**
κ / -	bare	2.91	1.72	1.25
	AuNC	0.048	3.00	1.48

S6.3. The SECM maps obtained for each studied system

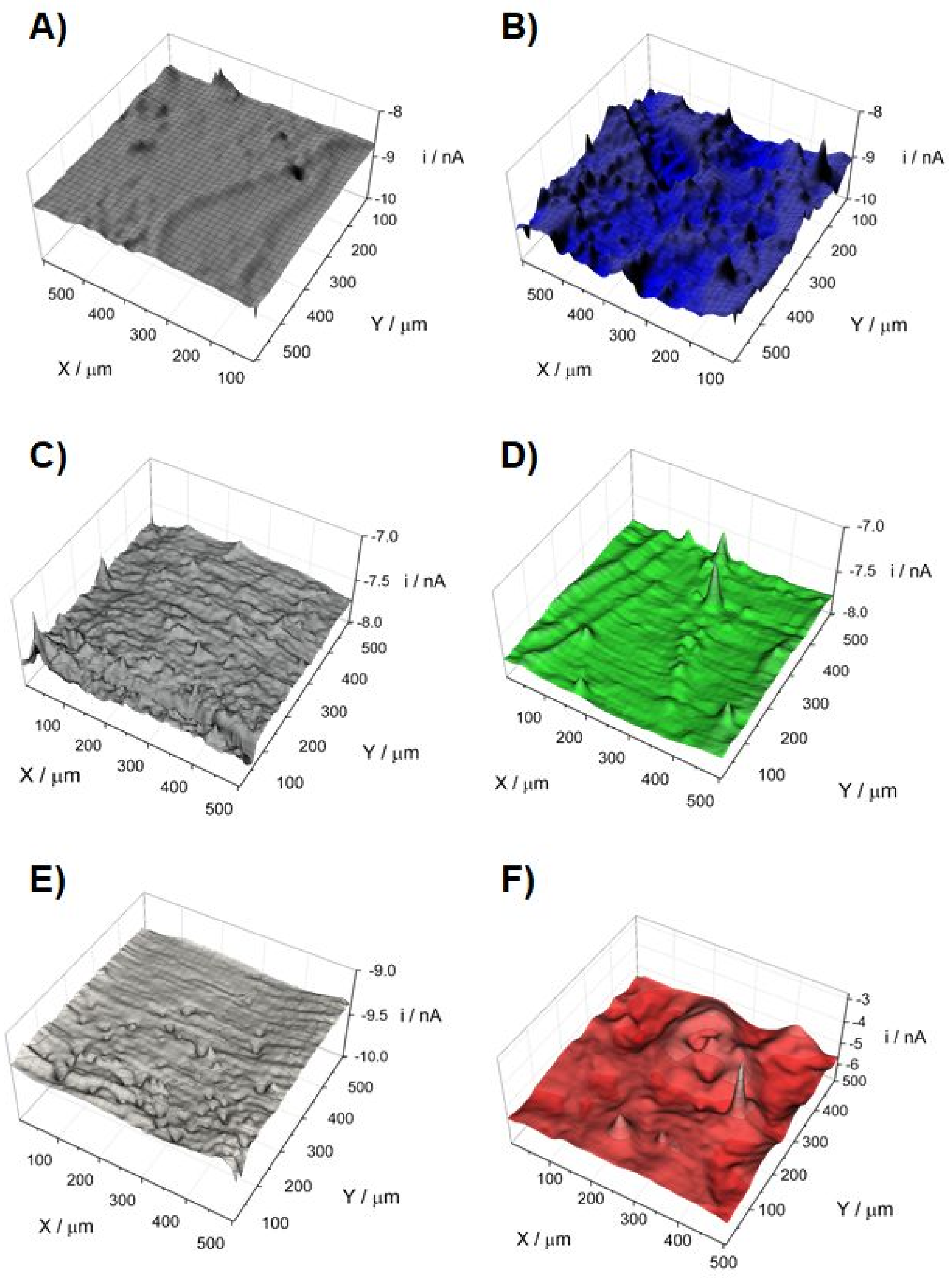

Fig S5 – SECM maps for different systems: A,B) GC; C,D) ITO; E,F) Au, characterizing the electrochemical homogeneity A,C,E) of bare electrodes and B,D,F) after AuNCs drop-casting. For all measurements, the working distance of the microelectrode was 5 µm, step 5 µm, and speed 10 µm s^{-1}.

S7. Calculation of the optimal R-S fit for different Hausdorf dimensions

Table S3 - The values of the least-square function R^2 for R-S formula ($D_H = 2$) and at D_H dimension offering the highest R^2

	**GC**	**Au**	**ITO**
$D_H = 2$	0.9967	0.9735	0.9477
$D_H = 1.99$	0.9949		
$D_H = 1.90$		0.9896	
$D_H = 1.85$			0.9828

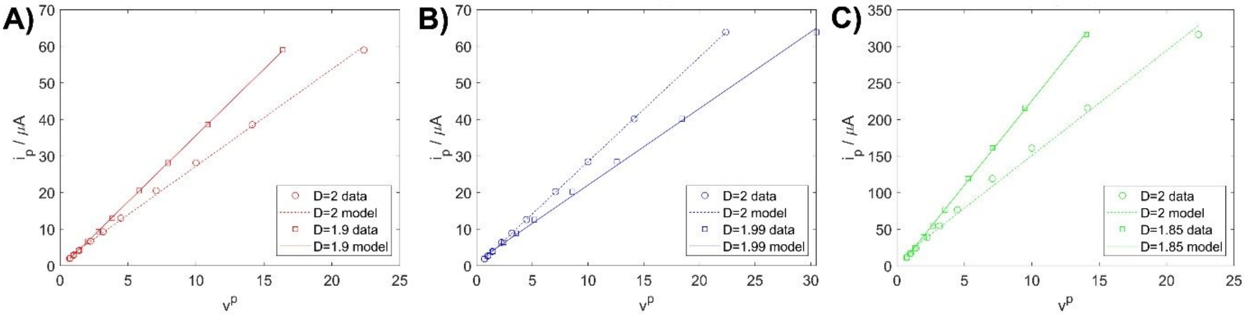

Fig S6 – Graphical representation of the Hausdorf dimension fitting after AuNCs drop-casting at each electrode surface: A) AuNC/Au; B) AuNC/GC; C) AuNC/ITO.

References:

(S1) Cornut, R.; Lefrou, C. New Analytical Approximation of Feedback Approach Curves with a Microdisk SECM Tip and Irreversible Kinetic Reaction at the Substrate. *Journal of Electroanalytical Chemistry* **2008**, *621* (2), 178–184. https://doi.org/10.1016/j.jelechem.2007.09.021.
